# Aqueous Ammonium Nitrate Investigated Using Photoelectron Spectroscopy of Cylindrical and Flat Liquid Jets

**DOI:** 10.1021/acs.jpcb.4c01755

**Published:** 2024-07-08

**Authors:** Tamires Gallo, Georgia Michailoudi, Joana Valerio, Luigi Adriano, Michael Heymann, Joachim Schulz, Ricardo dos Reis Teixeira Marinho, Flavia Callefo, Noelle Walsh, Gunnar Öhrwall

**Affiliations:** † Synchrotron Radiation Research, Lund University, Box 118, SE-22100 Lund, Sweden; ‡ MAX IV Laboratory, Lund University, Box 118, SE-22100 Lund, Sweden; § Nano and Molecular Systems Research Unit, University of Oulu, P.O. Box 3000, FI-90014 Oulu, Finland; ∥ European XFEL, Holzkoppel 4, Schenefeld 22869, Germany; ⊥ IBBS, Institut für Biomaterialien und Biomolekulare Systeme, Universität Stuttgart, Pfaffenwaldring 57, 70569 Stuttgart, Germany; # Institute of Physics, Brasilia University (UnB), 70.919-970 Brasiliá, Brazil; ∇ Institute of Physics, Federal University of Bahia, 40.170-115 Salvador, BA, Brazil; ⊗ Brazilian Synchrotron Light Laboratory, LNLS, Brazilian Center for Research in Energy and Materials, CNPEM, CP 6192, 13085-970 Campinas, SP, Brazil

## Abstract

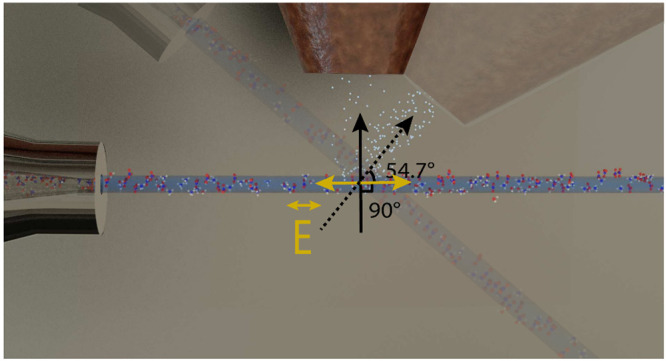

Ammonium nitrate in aqueous solution was investigated with synchrotron radiation based photoelectron spectroscopy using two types of liquid jet nozzles. Electron emission from a cylindrical microjet of aqueous ammonium nitrate solution was measured at two different angles relative to the horizontal polarization of the incident synchrotron radiation, 90° and 54.7° (the “magic angle”), for a range of photon energies (470–530 eV). We obtained β parameter values as a function of photon energy, based on a normalization procedure relying on simulations of background intensity with the SESSA (Simulation of Electron Spectra for Surface Analysis) package. The β values are similar to literature data for O 1s ionization of liquid water, and the β value of N 1s from NH_4_^+^ is higher than that for NO_3_^–^, by ≈0.1. The measurements also show that the photoelectron signal from NO_3_^–^ exhibits a photon energy dependent cross section variation not observed in NH_4_^+^. Additional measurements using a flat jet nozzle found that the ammonium and nitrate peak area ratio was unaffected by changes in the takeoff angle, indicating a similar distribution of both ammonium and nitrate in the surface region.

## Introduction

Due to its inherent surface and chemical sensitivity, photoelectron spectroscopy is an established tool for the investigation of the surface properties of a wide range of samples.^1^ It has frequently been used for quantitative analysis, where a full understanding of measurement data requires knowledge of, e.g., partial cross sections and elastic/inelastic scattering processes.^1^ Since the late 1990s, aqueous solutions, in particular, have been extensively studied using the liquid microjet technique^2,3^ and more recently, the measurement of photoelectron angular distributions (PADs) from a solution is of particular interest to this research field.

For free molecules in the gas phase, PAD measurements are relatively straightforward to interpret as the angular distribution of emitted photoelectrons is determined by the polarization of the incident light and the character of the molecular orbitals, with little influence from the surrounding environment. The photoelectron differential cross section for single-photon absorption of linearly polarized light^4^ is given by
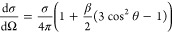
1where σ is the total photoionization cross section, Ω is the solid angle of detection, β is the anisotropy parameter, and θ is the angle between the polarization direction and the emitted electron. eq 1 has been widely applied to gas-phase atoms and (randomly oriented) molecules^5^ and even free clusters (due to the random orientation of the sample) to characterize the angular distribution of photoelectrons. Studies of free rare-gas clusters provide insights into angular distribution effects that one can expect in the condensed phase; a more anisotropic angular distribution of electrons from surface species has been observed compared to that from the bulk. This is due to the fact that elastic scattering of electrons from atoms in the surface of the cluster is less likely than from atoms in the bulk (the outgoing electron wave is more likely to interact with the surroundings as it leaves the bulk).^6−8^

The case for liquids is, however, even more complicated because the surface of the liquid may introduce an orientation of the sample, and the distribution of a solute is often nonuniform close to the surface. As in the case of clusters, the probability of electron scattering processes is lower for molecules close to the surface than in the bulk, leading to a more pronounced angular anisotropy for photoelectrons emitted by solutes with a propensity for the surface than those emitted by solutes that avoid the surface. In the case of a preferred orientation of the molecules at the surface, eq 1 would no longer be valid. The angular distributions from individual molecules would be richer in structure and different from those expected from eq 1,^5,9,10^ but averaged over a nonrandom distribution and affected by elastic scattering effects. Depending on the measurement geometry, such effects will thus have a substantial influence on the measured photoelectron intensities, and, thereby, the inferences regarding the surface propensity drawn from the data.^1^

The photoelectron angular distributions from valence and core orbitals in liquid water have been studied by several authors,^11−14^ and one general observation is that the anisotropy is decreased for the condensed liquid phase compared to the gas phase. The angular distributions of electrons emitted by solutes have, however, been less studied. In recent work, Dupuy et al.^15^ investigated the adsorption of octanoic acid at the liquid–vapor interface of aqueous solutions, using experimentally measured PADs to determine the relative depth/location of the carboxylate COO^–^ and carboxylic acid COOH functional groups in solutions with varying pH. Their PAD data also clearly demonstrates that one can obtain information regarding the orientation of the molecules at the liquid–vapor interface.

In this work, we present angle-resolved measurements of photoelectron intensity following core-excitation of ammonium nitrate in an aqueous solution, using both cylindrical liquid microjet and flat liquid jet sample introduction systems. This particular sample was chosen for several reasons. First, the nitrogen atoms in the respective ions yield N 1s photoelectron features well separated in energy, making it easy to distinguish between them. However, they are still close enough in energy that differences in transport and instrument transmission due to variations in kinetic energy will not appreciably affect the comparison of the yields. Furthermore, there has been some debate in the literature regarding the distribution of the nitrate ions in the vicinity of the surface (summarized below), and our angle-resolved photoemission data should provide further insight regarding this question.

It is long known that both the NH_4_^+^ and NO_3_^–^ ions will tend to increase the surface tension of aqueous solutions,^16^ and in a simple classical picture, one would therefore expect that both ions avoid the liquid surface. Still, several authors have studied the surface propensity of the nitrate anion using molecular dynamics (MD) simulations and reached conflicting conclusions. Using Car–Parrinello MD simulations of a cluster and classical MD simulations of an extended slab system using a polarizable force field, Salvador et al.^17^ found that NO_3_^–^ clearly prefers interfacial over bulk solvation. On the contrary, Dang et al.,^18^ using MD simulations with a different treatment of polarizability, conclude that the probability of finding the nitrate anion at the aqueous interface is quite small. Thomas et al.^19^ similarly concluded that the nitrate anion resides primarily below the first few surface water layers and has only a small probability of being at the surface of the solution. More recently, Mosallanejad et al.^20^ have studied high-concentration solutions of ammonium nitrate using several MD models. Specifically, they find that the approach of ions to the interface and their separation differ significantly as predicted by the OPLS (Optimized Potential for Liquid Simulations) and the OPLS/ECC (Electronic Continuum Correction) models. For the former, ions repel from the interface and form a depletion layer with a thickness of approximately 5 Å. The separation between layers of ions is rather small, while for the latter, the ions display pronounced segregation, with nitrate anions preferring to place themselves at the interface (a plot of the number density derived from their data is shown in Figure S7 in the Supporting Information).

Interestingly, the experimental studies reported in the literature also lead to a variety of conclusions. Some recent studies of NH_4_NO_3_ in aqueous solution point to the fact that the nitrate anion and the ammonium cation have a propensity for the surface and the bulk, respectively.^21−23^ Weeraratna et al.^21^ investigated NH_4_NO_3_ aerosols with soft X-ray spectroscopy, using a sample solution of 0.5 mol/dm^3^ concentration. They collected photoelectron spectra at 430 and 440 eV, and observed a higher spectral signal of NO_3_^–^ than NH_4_^+^, which suggests that the nitrate ion has a greater tendency to be present at the surface compared with the ammonium ion. However, the ammonium N 1s signal overlapped with a signal that the authors attributed to ammonia or amide, which complicated the analysis. Tian et al. and Hua et al.^22,23^ used phase-sensitive sum-frequency vibrational spectroscopy to investigate the air/water interface of salt solutions and reported the observation of an electric double layer formed at the interface, which was understood to have been generated by surface-active nitrate anions and different counterions, among them the ammonium cation.

In contrast to this, from liquid microjet photoelectron spectroscopy measurements on NaNO_3_ and NaNO_2_ solutions at high concentration (3.0 mol/dm^3^), Brown et al.^24^ concluded a preference for nitrate anions for bulk solvation in aqueous solution. In that analysis, the authors relied on the relative intensities of the O 1s and N 1s signal in binary aqueous solutions of NaNO_3_ and NaNO_2_, and a ternary aqueous solution with both NaNO_3_ and NaNO_2_, as well as the kinetic energy dependence of these quantities. This is a less immediate method than those applied in our current study. Additionally, the concentration is higher than that used in our study, and this could very well affect surface composition.

Clearly, there is no complete consensus on the surface propensity of the nitrate ion in aqueous solution. The sensitivity to the choice of model implemented in theoretical studies as well as the various conflicting conclusions from experimental studies, motivates further experimental data on this issue and a photoelectron spectroscopy study of NH_4_NO_3_, where direct comparison of NH_4_^+^ and NO_3_^–^ is possible, promises to yield valuable information that can assist in answering the many open questions. The recently developed flat jet nozzle used in this work affords us the possibility to vary the takeoff angle of the photoelectrons, thus allowing us to change the probing depth of the measurement to investigate the surface propensity of the ammonium and nitrate ions.

## Experimental Methods

**Figure 1:**
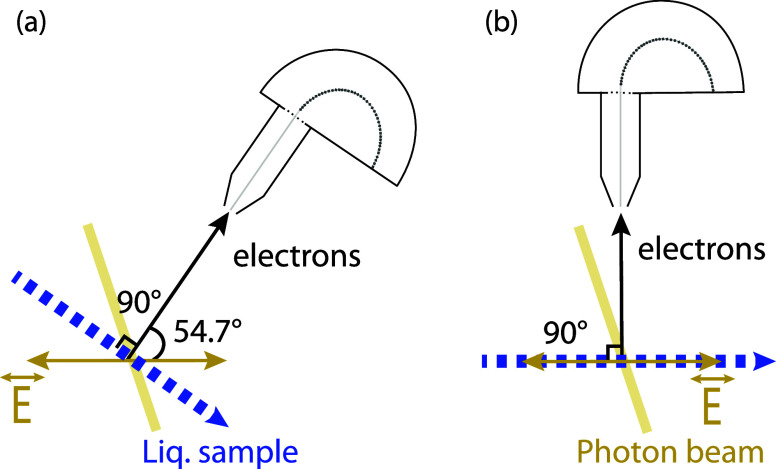
Schematic of experimental geometry at FlexPES beamline.

The experiments were performed using the Low Density Matter (LDM) photoemission endstation at the FlexPES beamline^25^ on the 1.5 GeV ring at MAX IV laboratory in Lund, Sweden. The endstation is equipped with a Scienta R4000 electron spectrometer, which can rotate around the photon beam in the plane perpendicular to it, allowing angle-resolved photoelectron spectroscopy measurements (Figure 1). The liquid sample, a 1.0 M (mol/dm^3^) aqueous solution of NH_4_NO_3_, was introduced as a jet perpendicular to both the photon beam and the spectrometer lens axis.

For the experiments where the angle with respect to the polarization vector and photon energy was varied, a cylindrical jet formed by a quartz nozzle was used. The nozzles that were used had a diameter of 22 and 25 μm (Advanced Microfluidic Systems GmbH). The liquid jet was collected in a liquid-nitrogen-cooled trap such that it was frozen after the interaction with the synchrotron radiation. A differentially pumped compartment was used to separate the low vacuum region where the liquid jet flows from that in the spectrometer chamber, where a vacuum of ≈2e–5 mbar or lower must be maintained to allow operation of the spectrometer. The liquid jet intersected the radiation from the beamline 2–3 mm after leaving the nozzle, well before the jet breaks up into droplets.

For the flat jet experiments, where the takeoff angle toward the spectrometer was varied, a 3D printed nozzle that was recently developed at EuXFEL,^26^ similar to that presented in,^27,28^ was used. Helium gas flows around the liquid sample causing the formation of a series of flat liquid sheets. The first flat sheet had a length ≈500 μm and width ≈300 μm, with the jet then collapsing to form a second flat sheet with smaller size in the perpendicular direction, and then consecutive sheets with decreasing size. Details about that system are presented in a separate paper.^26^ The measurements reported here were recorded using the first sheet immediately after the nozzle opening. The rod holding the nozzle was mounted on a rotary stage, allowing the angle between the liquid sheet surface and the incoming radiation to be varied. Thus, the takeoff angle for the electrons going toward the spectrometer mounted perpendicular to the photon beam could be varied. Other experimental conditions were kept the same as for the cylindrical jet. The flat sheet generated in this way has micrometer thickness, high stability, and optical flatness, important characteristics for spectroscopic studies on samples of aqueous solutions.^29,30^ The thicker rim that forms at the edges of the sheet prevents the investigation of very small takeoff angles.^27,31^ However, for angles ≥10°, we do not expect that the thicker rim will influence that data. Thus, we have limited our measurements to angles that are larger than this. Electron-gas collisions in the He gas flowing around the liquid sheet may, to some degree, influence the intensities measured with the electron spectrometer. However, the attenuation in the gas will not differ appreciably between the N 1s features from NH_4_^+^ and NO_3_^–^, and since we will only consider the ratio of the intensities, this will not affect the comparison with measurements recorded using the cylindrical jet.

As part of the analysis of the angle-resolved measurements where the angle with respect to the polarization was varied, we have used a normalization procedure for the data by comparing the background on-top of which the N 1s photoelectron lines sit (discussed below). The background in the kinetic energy range of the N 1s lines comes from scattered electrons originating from valence band photoionization or Auger decay of the N 1s core hole. To model the situation, we have used the software program SESSA (Simulation of Electron Spectra for Surface Analysis)^32^ to simulate electron spectra from aqueous solutions of NH_4_NO_3_. In the simulation the β values and kinetic energies of the primary electrons that end up in the background (the valence band photoelectrons or Auger electrons) may deviate somewhat from those for the real system, but they represent reasonable assumptions. Additionally, from the randomization of the (elastic and inelastic) scattering events, the outcome for background can be expected to be relatively insensitive to the initial parameters, which is why we believe it is a meaningful comparison.

Further details on experimental conditions and data analysis, including the simulations performed with SESSA, can be found in the Supporting Information.

## Results and Discussion

**Figure 2:**
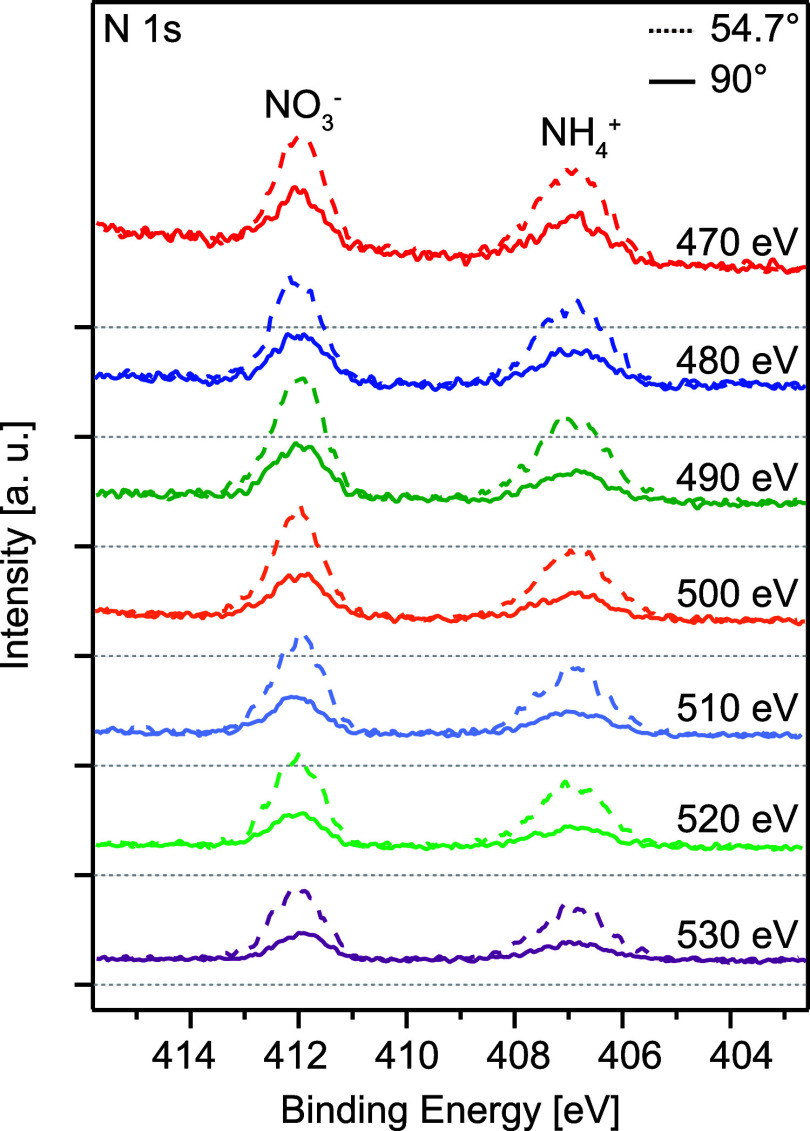
N 1s spectra from ammonium nitrate recorded at 54.7° (dashed line) and 90° (solid line), with photon energies at 470, 480, 490, 500, 510, 520, and 530 eV. The light gray dotted lines below each spectrum refer to the zero levels of their backgrounds.

In Figure 2, N 1s photoelectron spectra of aqueous NH_4_NO_3_ are shown, for photon energies ranging from 470 to 530 eV in steps of 10 eV and at two emission angles with respect to the horizontally polarized radiation, θ = 54.7° (dashed lines) and 90° (solid lines). The peaks located at 406.9 and 412.0 eV are assigned to NH_4_^+^, and NO_3_^–^, respectively. The energies are in good agreement with what has been reported for NO_3_^–^ in aqueous solutions of NaNO_3_ and HNO_3_^24,33^ and NH_4_^+^ in aqueous solutions of NH_4_Cl and (NH_4_)_2_SO_4_,^34,35^ but slightly higher than those reported by Weeraratna et al. for NH_4_NO_3_ in aerosol particles.^21^ The NH_4_^+^ peak is considerably broader than the NO_3_^–^ peak; the Gaussian widths were ≈1.4 eV for the NH_4_^+^ peak, slightly narrower than that reported for NH_4_Cl,^35^ and ≈1.0 eV for the NO_3_^–^ peak.

The peaks sit on top of a substantial background, the zero level of which is indicated by a gray dotted line below each spectrum in Figure 2. The spectra have been normalized for acquisition time (i.e., number of sweeps), and at each energy, the spectrum recorded at 54.7° has been scaled by a factor given by the ratio of the areas below the linear backgrounds used in the fitting procedure at 54.7° and 90°, such that the background levels coincide. The ratio background-intensity-90°:background-intensity-54.7° lies in the range of 0.84–0.95 for all photon energies, indicating a relatively high degree of constancy between the data sets recorded at the two angles. The N 1s peaks are more intense relative to the background in the spectra recorded at 54.7°, which is consistent with a positive value of the β parameter. With this normalization, a general decrease in intensity of the N 1s peaks with increasing photon energy can be seen, as expected from the decreasing N 1s photoionization cross section with increased photon energy and the reduced transmission of the spectrometer with increased kinetic energy. This latter effect is due to the fact that the spectrometer was operated with a fixed analyzer energy (pass energy), and the lens mode used had constant linear magnification. The physical angular acceptance into the analyzer in the dispersive direction is by design limited at the entrance slit of the analyzer, and, as a consequence of Liouville’s principle, the accepted angle from the sample will, therefore, be reduced when the ratio of the original electron kinetic energy and the pass energy is increased. The transmission of the instrument thus decreases with increasing kinetic energy.

**Figure 3:**
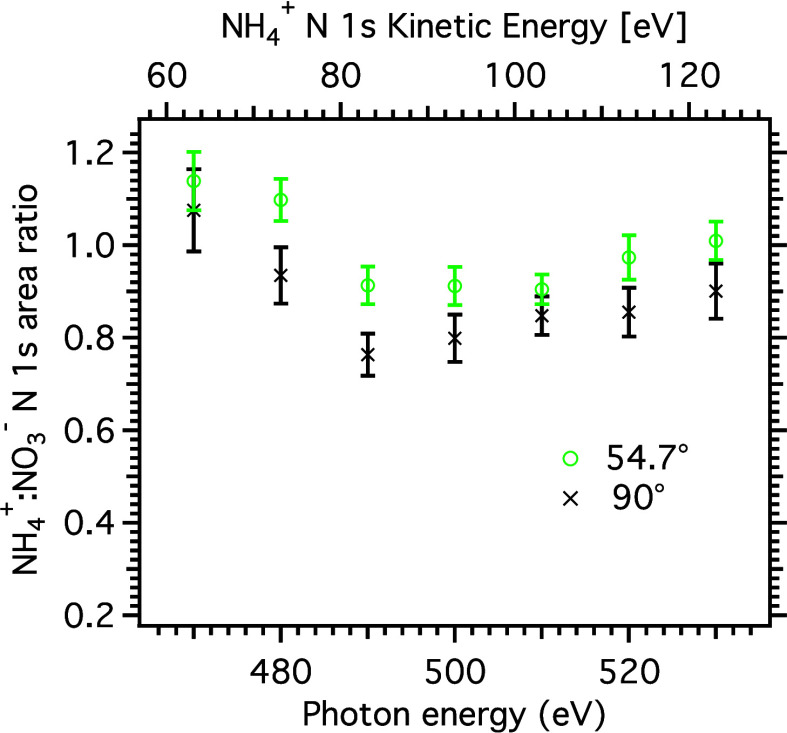
NH_4_^+^:NO_3_^–^ peak-area ratio obtained from N 1s spectra recorded at different photon energies, and at two angles of emission relative to the polarization of the radiation (54.7° and 90°). The corresponding kinetic energy for N 1s electrons from NH_4_^+^ is indicated by the top axis, the kinetic energy for the electrons from NO_3_^–^ is ≈5 eV lower. The error bars only include statistical uncertainties. Tabulated data are available in the Supporting Information.

The spectra have been fitted to derive the area of the peaks (an example fit and obtained areas normalized to acquisition time can be found in the Supporting Information, section Experimental data fitting, Figures S3 and S4). In Figure 3 the ratio of the area of the N 1s XPS peaks of the ammonium and nitrate ions as a function of photon energy for the angles 90° and 54.7° are shown. For both angles, the NH_4_^+^:NO_3_^–^ peak area ratio has a photon energy dependent variation: It decreases from 470 eV until a local minimum at 490–500 eV, after which the ratio increases up to 530 eV. The inelastic mean free path (IMFP) of the electrons is kinetic energy dependent, typically considered to have a minimum around 50–100 eV, and this observation in the range ≈60–125 eV could therefore be interpreted as being due to a difference in the distribution of the ions in the surface region and a minimum in the IMFP. Assuming that the minimum in the NH_4_^+^:NO_3_^–^ N 1s area ratio corresponds to the minimum IMFP this would then indicate that NO_3_^–^ ions reside closer to the surface than the NH_4_^+^ ions, in accordance with the findings of some authors.^20−23^ However, the IMFP for the electrons is not expected to differ much for peaks that are so close in kinetic energy, and we, therefore, conclude that the interpretation of that variation as being due to a minimum in the IMFP is unlikely. Quantitatively, the effective attenuation length (EAL) in liquid water has been estimated to vary from ≈1 nm at to ≈1.5 nm between 60 and 130 eV,^12^ or to have a nearly constant value around ≈2 nm^36,37^ in this range, corresponding to slightly larger IMFP:s.

The fact that the ratio has a value larger than the stoichiometric value 1 for some energies in the data recorded at 54.7°, where angular distribution effects should be negligible, also speaks against this interpretation. At higher kinetic energies, the ratio seems to tend toward 1 in the data from 54.7°, which could be an indication that NO_3_^–^ and NH_4_^+^ ions have a similar depth profile, but considering the observed variation of the ratio in the studied energy range, this is inconclusive.

The oscillatory behavior of the NH_4_^+^:NO_3_^–^ N 1s peak area ratio is observed at both angles and for the 54.7° recordings, angular distribution effects should not affect this ratio. Variations in the IMFP together with a difference in the depth distribution of the ions could in principle cause such an effect, but since the IMFP has been shown to be relatively flat in this kinetic energy region,^12^ the explanation has to be sought elsewhere.

Experimental N 1s Near-Edge X-ray Absorption Spectra together with theoretical modeling have been used to investigate the structure of bulk solvation for aqueous ammonium^38−40^ and nitrate^41^ ions. Unfortunately, the photon energy ranges used in those studies (≈ 400–425 eV) did not extend to the range studied here. Furthermore, there could possibly be differences in the solvation for the ions in the near-surface region observed using surface-sensitive photoelectron spectroscopy measurements. Additionally, since our photoelectron spectroscopy measurements are more surface-sensitive than the transmission^38,39^ and total electron yield (TEY)^41^ XAS measurements, there could possibly be differences due to variations in the solvation for the ions in the near-surface region.

**Figure 4:**
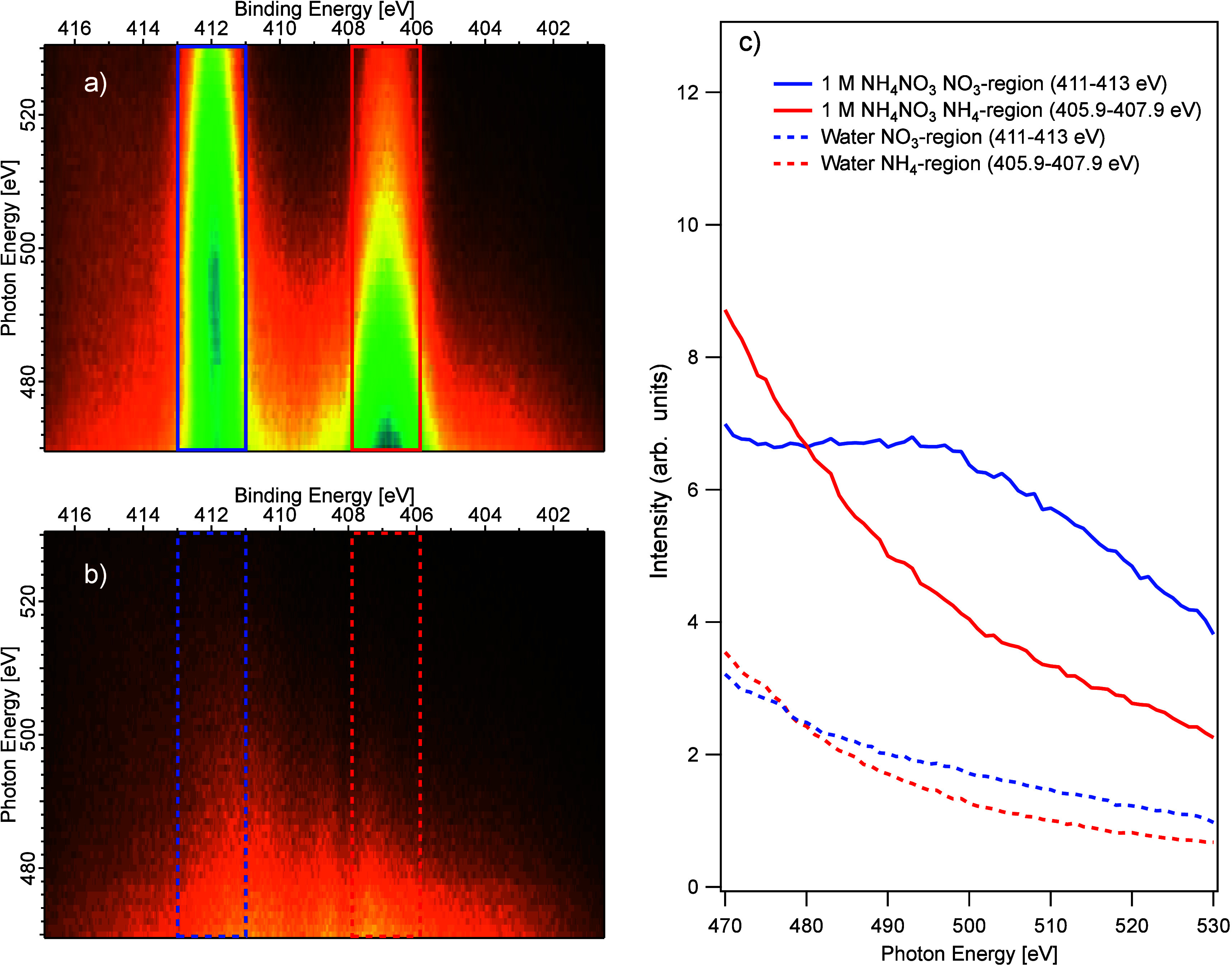
Left: Electron yield maps of the N 1*s* region recorded in “fixed mode” (see text), for photon energies between 470 and 530 eV, with 1 eV step, recorded at 54.7° to the polarization vector. At the top left [a)], data for a 1.0 M NH_4_NO_3_ aqueous solution are shown, and at the bottom left [b)] data for a 25 mM NaCl aqueous solution. The integrated intensity within ranges that encompass the N 1*s* peaks are plotted in the graph on the right [c)] (blue solid line: NO_3_^–^ in the NH_4_NO_3_ solution, red solid line NH_4_^+^, blue and red dashed lines: corresponding energy ranges in the NaCl solution). Note the oscillation in the NO_3_^–^ N 1*s* peak intensity, which is absent for the other cases.

To investigate the observed oscillation further, we have recorded photoelectron spectra of the N 1*s* region of aqueous NH_4_NO_3_ over the same energy range with finer energy steps (1 eV), using the so-called “fixed” mode of the spectrometer. In “fixed” mode, the spectrum is acquired by accumulating data only for the range of energies that simultaneously hit the detector (≈ ±4% of the pass energy, ≈ ±8 eV for the 200 eV pass energy used), without changing the accelerating (or retarding) potentials needed to bring the electrons to the detector. This speeds up the acquisition, however, variation of transmission through the spectrometer and of the sensitivity of the detector will affect the linearity of the response for the range of kinetic energies hitting the detector. Note that in the normal “swept” mode, used for all the other measurements presented in this paper, the spectrum is recorded by changing the accelerating potential, so that all parts of the spectrum have been swept across the detector, and the variations observed in the “fixed” mode are canceled out. In Figure 4, the raw data from this recording is presented as a color map, with blue as the highest intensity, and black as the lowest (top left). The N 1*s* lines are visible, and a clear oscillatory behavior can be seen in the intensity of the NO_3_^–^ peak, which is not seen for the NH_4_^+^ peak. To make sure that the variation in intensity is not due to the kinetic energy dependence of the transmission of the spectrometer, we also recorded spectra for the same range of kinetic energies for a solution that does not contain any nitrogen atoms, namely a 25 mM NaCl solution, which is shown at the bottom. For this energy range, a nearly flat background is expected, and the nonuniform intensity reflects the inhomogeneity of the detector and transmission variations. The absence of any oscillatory behavior as a function of photon energy shows that the observation for the NO_3_^–^ peak is due to the sample itself and not the spectrometer. A similar behavior for N 1s of NO_3_^–^ was also observed in the “swept” mode spectra in both the 54.7° and the 90° measurements (see Figure S4, Supporting Information), strengthening the conclusion that it is the photoionization cross section of NO_3_^–^ that is varying in an oscillatory fashion.

**Figure 5:**
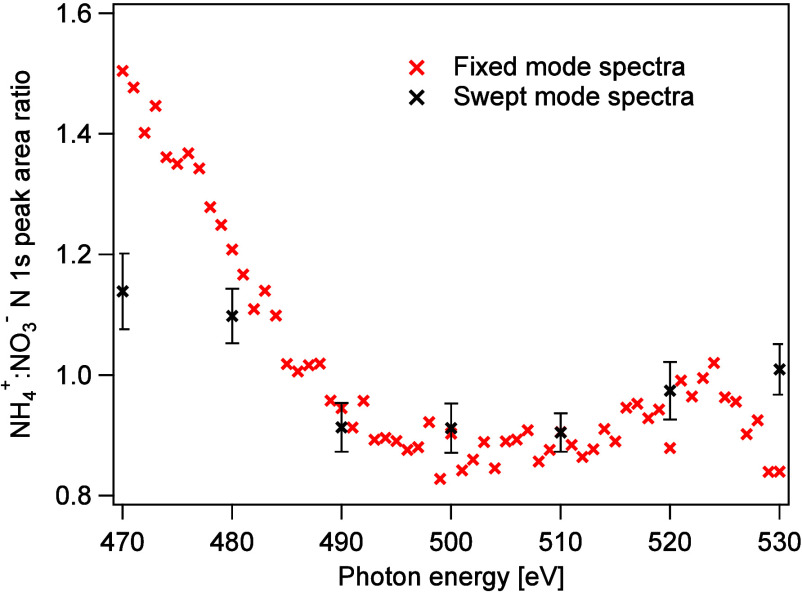
NH_4_^+^:NO_3_^–^ peak-area ratio obtained from the N 1s spectra in Figure 4, after being normalized to the data from the 25 mM NaCl solution to compensate for transmission variations and uneven sensitivity over the detector. The data for swept mode spectra for 54.7° from Figure 3 are also included.

To obtain quantitative data, we have attempted to compensate for the variations in transmission and detector inhomogeneity by normalizing the data from the 1.0 M NH_4_NO_3_ solution with the data for the NaCl solution, recorded with the same experimental parameters. Example spectra are shown in the Supporting Information (Figure S5). This procedure relies on the assumption that for the NaCl solution the spectrum should not have any structure and that the variation in the observed intensities reflects only the spectrometer transmission and the detector inhomogeneity. We have fitted these normalized data and the obtained NH_4_^+^:NO_3_^–^ peak-area ratio as a function of photon energy is shown in Figure 5, where the swept mode data for 54.7° recordings from Figure 3 are also included for comparison. The overall trends of the two data sets are similar, with the ratio being larger than 1 in the lower energy range, becoming lower than 1 just below 490 eV, and then possibly increasing slightly, approaching 1 as the photon energy increases. However, the numeric agreement is poor, especially in the lower photon energy range, and we attribute this to inadequacies of the normalization procedure for the “fixed” mode data. We have abstained from presenting error bars for the “fixed” mode data, as we believe that the systematic errors are larger than the statistical ones. However, an indication of the statistical uncertainty can be obtained from the point-to-point variation of the ratio.

As the investigated energy region is so far into the continuum that shape resonances or other near-threshold processes hardly can come into play, we tentatively interpret the clear observation of the oscillation in the NO_3_^–^ N 1*s* intensity in the finer grid “fixed” mode as evidence that the NO_3_^–^ either has a more defined solvation structure than NH_4_^+^, giving rise to EXAFS (Extended X-ray Abosrption Fine Structure) oscillations, or that intramolecular scattering of the type earlier observed for chlorinated ethane molecules in the gas phase^42,43^ and trichloroethanol in aqueous solution^44^ occurs for NO_3_^–^ and affects the photoionization cross section.

Figure 3 illustrates the need for caution when drawing conclusions from the photoelectron peak area ratios, since, depending on which data point the analysis is based on, it is possible to arrive at opposing conclusions. For example, at 480 eV the ratio is above 1 for 54.7° and below 1 at 90°. In other words, at this particular photon energy, the data recorded at 54.7° seems to indicate that the ammonium molecules are more prominent at the surface than nitrate, whereas the opposite applies at 90°, where that data seems to show a higher nitrate concentration at the surface than ammonium. Clearly, the angular distribution affects the quantitative information and the inferences drawn from the data.

**Figure 6:**
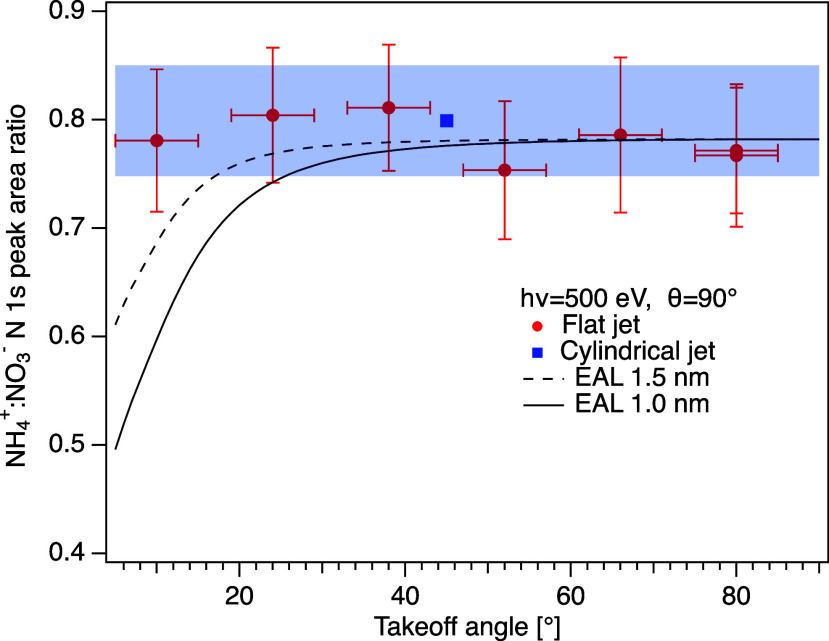
NH_4_^+^:NO_3_^–^ peak-area ratio obtained from N 1s spectra recorded with a flat jet as a function of takeoff angle from the liquid surface (red filled circles), at 500 eV photon energy and with a 90° angle between the polarization vector and the spectrometer lens axis. Tabulated data are available in the Supporting Information. The value obtained with the cylindrical jet, which will have contributions from all possible takeoff angles, at the same photon energy and angle, is also included (same data point as in Figure 2, blue filled square, arbitrarily positioned at 45°). The shaded light blue area indicates the range of the error bars for the cylindrical jet. Also included are estimates of the expected signal ratio based on MD simulations by Mosallanejad et al.^20^ (see text).

To obtain greater insight into the surface propensity of the nitrate and ammonium ions, we investigated the signal intensity as a function of takeoff angle, using a flat jet nozzle (example spectra shown in the Supporting Information, Figure S6). The distance traveled by the electrons inside the liquid will be larger for small takeoff angles, thereby enhancing the relative contribution from the surface layers. In Figure 6, the ratio of NH_4_^+^:NO_3_^–^ N 1s peak areas obtained from measurements of a 1.0 M NH_4_NO_3_ aqueous solution are presented, as a function of the takeoff angle from the liquid surface. The spectra were recorded at a photon energy of 500 eV, and the angle between the polarization vector and the lens axis was set to 90°. In the range of 10°–80°, we do not see any significant dependence on the takeoff angle, which indicates a similar distribution of the NH_4_^+^ and NO_3_^–^ ions in the near-surface region. The value obtained with the cylindrical jet under the same conditions matches the data very well. Estimates of the ratio of the ion signals, based on density profiles obtained from MD simulations for the case of a 2.5 m (mol/kg, corresponding to ≈2.25 M, calculated using the density obtained with the OPLS/ECC model from ref.^20^) aqueous solution of NH_4_NO_3_,^20^ and EAL:s of 1.0 and 1.5 nm (similar to experimentally determined values around 100 eV kinetic energy^12^) are also included in Figure 6 (details regarding the derivation of this estimate can be found in the Supporting Information material, section 3). As can be seen, for small takeoff angles the measured ratio deviates substantially from that estimated by this model. The concentration used for the MD simulation was 2.5 m, considerably higher than what we have used in our measurements (1 M), but simulations for 0.65 m solutions were reported to show similar behavior.^20^ Our results thus indicate a smaller difference in the distribution of the two ions and speak against a strong tendency for NO_3_^–^ to reside closer to the surface than NH_4_^+^.

The NH_4_^+^:NO_3_^–^ N 1s peak area ratio shown in Figure 3 exhibits a clear difference between the two angles, being consistently higher at 54.7°. This clearly shows that there is a difference in the angular distribution for N 1s levels of the ammonium and nitrate ions. The intensity of electrons from ammonium is reduced more than those from nitrate at 90°, i.e. they have a more anisotropic distribution and a higher β value. To estimate a value of the anisotropy parameter β for the N 1s orbitals in NH_4_^+^ and NO_3_^–^ using the data recorded with the cylindrical nozzle, we first assumed that the ions were randomly oriented in the sample, and then used two approaches to normalize the data from the two angles. As discussed in the Introduction, there are contradictory reports in the literature regarding the surface propensity of the nitrate ion and if they were to reside at the surface, a preferential orientation could occur. Our flat jet data indicate that there is no substantial difference between the nitrate and ammonium ions as regards their propensity for the surface, and macroscopic surface tension measurements indicate that both avoid the surface.^16^ Thus, we assume that the ions avoid the immediate surface, and, therefore, cannot be expected to show any alignment with respect to it.

**Figure 7:**
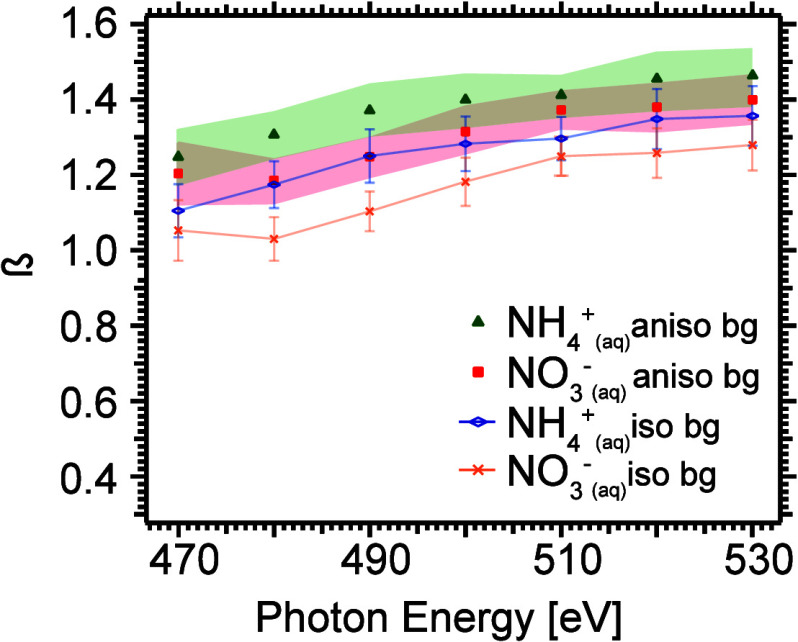
β value as a function of photon energy for NH_4_^+^ and NO_3_^–^, for a case assuming an isotropic background (NH_4_^+^ blue symbols and NO_3_^–^ yellow symbols), and an anisotropic background obtained from SESSA simulations (NH_4_^+^ green symbols and NO_3_^–^ red symbols). The estimated error (only including statistical uncertainties) is shown as bars in the first case and light-colored ranges in the second. Tabulated data are available in the Supporting Information.

The normalization of the data is based on the observation that the background in the photoelectron spectra derives from inelastically scattered electrons, coming from the same source volume as the N 1s electrons. In this case, the origin of the background is inelastically scattered valence electrons (from water and NH_4_NO_3_) and N 1s Auger electrons (from NH_4_NO_3_). Since these electrons (N 1s and background) have approximately the same kinetic energy, they will have very similar transmission through the spectrometer. By normalizing the N 1s signal to the background intensity, we will, therefore, remove the influence of geometric factors when changing the angle, such as changes in the overlap between the X-ray beam and liquid jet or changes in transmission of the spectrometer. However, a question remains as to whether the background itself is isotropic or not, and we have used two approaches in an attempt to answer that question. In the scattering processes (inelastic and elastic) there is a tendency to randomize the direction of the electrons, meaning that the background electrons will certainly have a more isotropic distribution than they originally had. The valence electrons of both water and NH_4_NO_3_ all have positive β values at the photon energies studied here, and the N 1s Auger electrons are expected to be nearly isotropic, which means that if the background electrons display an anisotropy in their angular distribution, it will be with a positive β value, substantially lower than that of the valence band electrons. One limiting case is when the background is assumed to be isotropic (β = 0), meaning that the background intensity at 54.7° and 90° would be immediately comparable. The data used for the plots in Figure 2 were normalized in this way, and from this we have determined β values for N 1s of NH_4_^+^ and NO_3_^–^ using eq 1. These are displayed as a function of photon energy in Figure 7 (blue and yellow symbols for NH_4_^+^ and NO_3_^–^, respectively).

Using this normalization, the experimentally determined β values for the N 1s levels are observed to increase from ≈1 at 470 eV to ≈1.3 at 530 eV, with the β value of NH_4_^+^ consistently being higher than that of NO_3_^–^, by ≈0.1. The error bars (estimated only by statistical uncertainties) for the two N 1s levels overlap, but the consistently higher value for NH_4_^+^ gives us confidence that it has a higher β value than NO_3_^–^. We note that these values are similar to those obtained for O 1s in liquid water, where the β value was found to increase from ≈1.0 to ≈1.4 in the kinetic energy range 60–120 eV.^12^ This result is not unexpected, since the solvent is the same in both cases, and N 1s and O 1s photoelectron angular distributions can be expected to be similar for the kinetic energies investigated here, as shown in calculations of 1s anisotropy parameters for gas-phase NH_3_ and H_2_O.^45,46^ Buttersack et al.^47^ determined a β value of 1.66 ± 0.26 for N 1s in liquid ammonia at 640 eV photon energy (≈ 235 eV kinetic energy), but the higher kinetic energy makes a direct comparison less relevant, and the electron scattering probabilities in liquid ammonia may well be different from those in water.

We have also simulated the photoelectron spectra of aqueous NH_4_NO_3_ for this range of photon energies using the software program SESSA (Simulation of Electron Spectra for Surface Analysis).^32^ In these simulations, database values are used for the cross sections and β parameter of the valence and Auger lines, as well as for the inelastic and elastic scattering probabilities in the sample. While these may not match the experimental reality perfectly, they will almost certainly yield a better description than the simple assumption of an isotropic angular distribution for the background electrons used in the first approach. Using several different approaches for the distribution of the solute at the surface (see the Supporting Information material (SI section 1)), we obtained a β value of ≈0.3 for the background electrons in the vicinity of the N 1s photoelectron peaks, nearly independent of photon energy and sample distribution model. This small positive value of the β value for the background means that the scaling used in the simple approach with an isotropic background shown in Figure 2 overemphasizes the intensity at 90°, and, therefore, the 90° spectra should be reduced in intensity, thus leading to higher β values for the N 1s lines. In Figure 7, the β values based on the normalization to the background intensity from the SESSA simulation are shown as green and red symbols for NH_4_^+^ and NO_3_^–^, respectively, and they are ≈0.15 higher than those derived from the assumption of an isotropic distribution for the background electrons.

## Conclusions

We have investigated the angular distribution of N 1s photoelectrons for a 1.0 M aqueous solution of ammonium nitrate, using a cylindrical liquid jet setup. Spectra were recorded at 54.7° and 90° relative to the horizontal polarization of the synchrotron radiation, with photon energies ranging from 470 to 530 eV.

The NH_4_^+^:NO_3_^–^ N 1s peak ratio recorded at 54.7 varies around 1 in this energy range, and we attribute the observed variation to EXAFS-like oscillations in the N 1s photoionization cross section, and our measurements indicate that it is the N 1s intensity of the NO_3_^–^ ion that mainly varies. We speculate that it may be due to a more defined solvation shell for nitrate, or that the electrons from the nitrogen atom in nitrate are more strongly scattered by the oxygen atoms than those in ammonium by the hydrogen atoms. The fact that the NH_4_^+^:NO_3_^–^ N 1s peak ratio is consistently higher at 54.7° than at 90° shows that the N 1s electrons from NH_4_^+^ have a higher β value than those from NO_3_^–^.

We have also recorded surface-sensitive N 1s spectra using a flat jet nozzle and varied the takeoff angle to investigate the surface propensity of the ions. The data indicate that NO_3_^–^ and NH_4_^+^ have similar distributions in the surface region, which speaks against NO_3_^–^ having a propensity for the surface, as has been suggested by other authors.^20−23^

Using a procedure where the N 1s intensities are normalized to background intensity, we have determined the anisotropy parameter β from the experimental data. Two approaches were used; one where an isotropic distribution was assumed for the background electrons, and one where simulations with the SESSA software^32^ were used to estimate the anisotropy of the background electrons, which yielded the result of β ≈ 0.3 for the background electrons. With the assumption of an isotropic background, the β value was found to be ≈1.0 at 470 eV, increasing to ≈1.25 at 530 eV for NO_3_^–^, and consistently higher by ≈0.1 for NH_4_^+^. Using the other approach, both these values increased by ≈0.15. Similar anisotropy parameter values have been observed for O 1s in liquid water in the same kinetic energy range,^12^ which is not unexpected since the same solvent is used and N 1s and O 1s can be expected to have similar behavior regarding the angular anisotropy.

In conclusion, we note that the fact that the N 1s of NH_4_^+^ has a higher β value than that of NO_3_^–^ results in a NH_4_^+^:NO_3_^–^ N 1s peak ratio which can be substantially different at 90° and 54.7°, and even cause opposite inferences regarding the propensity for the surface of the ions to be drawn from the data. This shows the importance of taking angular distribution effects into account for quantitative analysis of liquid surfaces.

## Supporting Information Available

Further experimental details; Additional information and figures show the SESSA simulation parameters and fitting of example spectra for both cylindrical and flat jet nozzles; Tabulated data for N 1s peak area ratios NH_4_^+^:NO_3_^–^, for both cylindrical jet and flat jet delivery systems, and β values for NH_4_^+^ and NO_3_^–^ derived assuming an isotropic background as well as an anisotropic background from SESSA simulations; Additional information on the derivation of the takeoff angle dependence of the photoelectron signal ratios from MD simulations (PDF)

## Footnotes

1 In general, the intensity of the photoelectron signal from a species *i* leaving the surface of a condensed matter sample can be described by

2

where Φ is the photon flux, σ_*i*_ is the angle integrated cross section for species *i*, *f*_*i*_(θ, *hν*) describes the angular distribution (PAD), *S*(*E*_*k*_) is a function describing spectrometer transmission and detector efficiency, and *T*(*n*_*i*_(*z*), α, λ{*z*, *E*_*k*_}) is a function depending on *n*_*i*_, the number density of species *i* as a function of *z*, the distance from the surface, and the transport characteristics of the sample, which depend on α, the angle between the surface normal and the spectrometer, and the inelastic and elastic scattering properties, quantified by their mean free paths, λ_*inel*_{*z*, *E*_*k*_} and λ_*el*_{*z*, *E*_*k*_}, which generally depend on the constitution of the sample as a function of *z*. *E*_*k*_ = *hν* – *E*_*b*,*i*_ is the kinetic energy of the electron, where *E*_*b*,*i*_ is the binding energy of species *i*.

## References

[ref1] Hüfner S. Photoelectron Spectroscopy, Principles and Applications, 3rd ed.; Springer Verlag: Berlin Heidelberg New York, 2003.

[ref2] Faubel M.; Steiner B.; Toennies J. Photoelectron Spectroscopy of Liquid Water, Some Alcohols, and Pure Nonane in Free Micro Jets. J. Chem. Phys. 1997, 106, 9013–9031. 10.1063/1.474034.

[ref3] Winter B.; Faubel M. Photoemission from Liquid Aqueous Solutions. Chem. Rev. 2006, 106, 1176–1211. 10.1021/cr040381p. 16608177

[ref4] Cooper J.; Zare R. N. Angular Distribution of Photoelectrons. J. Chem. Phys. 1968, 48, 942–943. 10.1063/1.1668742.

[ref5] Dill D. Fixed-Molecule Photoelectron Angular Distributions. J. Chem. Phys. 1976, 65, 1130–1133. 10.1063/1.433187.

[ref6] Öhrwall G.; Tchaplyguine M.; Gisselbrecht M.; Lundwall M.; Feifel R.; Rander T.; Schulz J.; Marinho R. R. T.; Lindgren A.; Sorensen S. L.; et al. Observation of Elastic Scattering Effects on Photoelectron Angular Distributions in Free Xe Clusters. Journal of Physics B: Atomic, Molecular and Optical Physicss 2003, 36, 3937–3949. 10.1088/0953-4075/36/19/005.

[ref7] Berrah N.; Rolles D.; Pešic Z. D.; Hoener M.; Zhang H.; Aguilar A.; Bilodeau R. C.; Red E.; Bozek J. D.; Kukk E.; et al. Probing Free Xenon Clusters From Within. Eur. Phys. J. Spec. Top. 2009, 169, 59 10.1140/epjst/e2009-00973-0.

[ref8] Zhang C.; Andersson T.; Förstel M.; Mucke M.; Arion T.; Tchaplyguine M.; Björneholm O.; Hergenhahn U. The Photoelectron Angular Distribution of Water Clusters. J. Chem. Phys. 2013, 138, 234306 10.1063/1.4809748. 23802959

[ref9] Shigemasa E.; Adachi J.; Oura M.; Yagishita A. Angular Distributions of 1sσPhotoelectrons from Fixed-in-Space N_2_Molecules. Phys. Rev. Lett. 1995, 74, 359–362. 10.1103/PhysRevLett.74.359. 10058738

[ref10] Yagishita A.; Hosaka K.; Adachi J. Photoelectron Angular Distributions from Fixed-in-Space Molecules. J. Electron Spectrosc. Relat. Phenom. 2005, 142, 295–312. 10.1016/j.elspec.2004.09.005.

[ref11] Ottosson N.; Faubel M.; Bradforth S. E.; Jungwirth P.; Winter B. Photoelectron Spectroscopy of Liquid Water and Aqueous Solution: Electron Effective Attenuation Lengths and Emission-Angle Anisotropy. J. Electron Spectrosc. Relat. Phenom. 2010, 177, 60–70. 10.1016/j.elspec.2009.08.007.

[ref12] Thürmer S.; Seidel R.; Faubel M.; Eberhardt W.; Hemminger J. C.; Bradforth S. E.; Winter B. Photoelectron Angular Distributions from Liquid Water: Effects of Electron Scattering. Phys. Rev. Lett. 2013, 111, 173005 10.1103/PhysRevLett.111.173005. 24206487

[ref13] Nishitani J.; West C. W.; Suzuki T. Angle-Resolved Photoemission Spectroscopy of Liquid Water at 29.5 eV. *Structural*. Dynamics 2017, 4, 044014 10.1063/1.4979857. PMC5384855 28405592

[ref14] Gozem S.; Seidel R.; Hergenhahn U.; Lugovoy E.; Abel B.; Winter B.; Krylov A. I.; Bradforth S. E. Probing the Electronic Structure of Bulk Water at the Molecular Length Scale With Angle-Resolved Photoelectron Spectroscopy. J. Phys. Chem. Lett. 2020, 11, 5162–5170. 10.1021/acs.jpclett.0c00968. 32479725

[ref15] Dupuy R.; Filser J.; Richter C.; Seidel R.; Trinter F.; Buttersack T.; Nicolas C.; Bozek J.; Hergenhahn U.; Oberhofer H.; et al. Photoelectron Angular Distributions as Sensitive Probes of Surfactant Layer Structure at the Liquid–Vapor Interface. Phys. Chem. Chem. Phys. 2022, 24, 4796–4808. 10.1039/D1CP05621B. 35156668 PMC8865751

[ref16] Marcus Y. Surface Tension of Aqueous Electrolytes and Ions. J. Chem. Eng. Data 2010, 55, 3641–3644. 10.1021/je1002175.

[ref17] Salvador P.; Curtis J. E.; Tobias D. J.; Jungwirth P. Polarizability of the Nitrate Anion and its Solvation at the Air/Water Interface. Phys. Chem. Chem. Phys. 2003, 5, 3752 10.1039/b304537d.

[ref18] Dang L. X.; Chang T.-M.; Roeselova M.; Garrett B. C.; Tobias D. J. On NO_3_^–^ - *H*_2_*O*Interactions in Aqueous Solutions and at Interfaces. J. Chem. Phys. 2006, 124, 066101 10.1063/1.2171375. 16483244

[ref19] Thomas J. L.; Roeselova M.; Dang L. X.; Tobias D. J. Molecular Dynamics Simulations of the Solution-Air Interface of Aqueous Sodium Nitrate. J. Phys. Chem. A 2007, 111, 3091 10.1021/jp0683972. 17402716

[ref20] Mosallanejad S.; Oluwoye I.; Altarawneh M.; Gore J.; Dlugogorski B. Z. Interfacial and Bulk Properties of Concentrated Solutions of Ammonium Nitrate. Phys. Chem. Chem. Phys. 2020, 22, 27698 10.1039/D0CP04874G. 33242055

[ref21] Weeraratna C.; Kostko O.; Ahmed M. An Investigation of Aqueous Ammonium Nitrate Aerosols with Soft X-Ray Spectroscopy. Mol. Phys. 2022, 120, e1983058 10.1080/00268976.2021.1983058.

[ref22] Hua W.; Verreault D.; Allen H. Surface Electric Fields of Aqueous Solutions of NH_4_NO_3_, Mg(NO_3_)_2_, NaNO_3_, and LiNO_3_: Implications for Atmospheric Aerosol Chemistry. Journal of Physics Chemistry C 2014, 118, 24941–24949. 10.1021/jp505770t.

[ref23] Tian C.; Byrnes S. J.; Han H.-L.; Shen Y. R. Surface Propensities of Atmospherically Relevant Ions in Salt Solutions Revealed by Phase-Sensitive Sum Frequency Vibrational Spectroscopy. J. Phys. Chem. Lett. 2011, 2, 1946–1949. 10.1021/jz200791c.

[ref24] Brown M.; Winter B.; Faubel M.; Hemminger J. Spatial Distribution of Nitrate and Nitrite Anions at the Liquid/Vapor Interface of Aqueous Solutions. J. Am. Chem. Soc. 2009, 131, 8354–8355. 10.1021/ja901791v. 19530722

[ref25] Preobrajenski A.; Generalov A.; Ohrwall G.; Tchaplyguine M.; Tarawneh H.; Appelfeller S.; Frampton E.; Walsh N. FlexPES, a Versatile Soft X-Ray Beamline at MAX IV Laboratory. J. Synchrotron Rad. 2023, 30, 831–840. 10.1107/S1600577523003429. PMC10325024 37159290

[ref26] Gallo T.; Adriano L.; Heymann M.; Wrona A.; Walsh N.; Öhrwall G.; Callefo F.; Skruszewicz S.; Namboodiri M.; Marinho R. Development of a Flat Jet Delivery System for Soft X-Ray Spectroscopy at MAX IV. J. Synchrotron Rad. 2024. 10.1107/S1600577524006611 PMC11371042 39172090

[ref27] Koralek J. D.; Kim J. B.; Bruža P.; Curry C. B.; Chen Z.; Bechtel H. A.; Cordones A. A.; Sperling P.; Toleikis S.; Kern J. F.; et al. Generation and Characterization of Ultrathin Free-Flowing Liquid Sheets. Nat. Commun. 2018, 9, 1353 10.1038/s41467-018-03696-w. 29636445 PMC5893585

[ref28] Schulz J.; Bielecki J.; Doak R. B.; Dörner K.; Graceffa R.; Shoeman R. L.; Sikorski M.; Thute P.; Westphal D.; Mancuso A. P. A Versatile Liquid-Jet Setup for the European XFEL. J. Synchrotron Radiat. 2019, 26, 339–345. 10.1107/S1600577519000894. 30855241 PMC6412181

[ref29] Chang Y.; Yin Z.; Balciunas T.; Worner H. J.; Wolf J. Temperature Measurements of Liquid Flat Jets in Vacuum. Struct. Dyn. 2022, 9, 014901 10.1063/4.0000139. 35224132 PMC8853733

[ref30] Galinis G.; Strucka J.; Barnard J. C. T.; Braun A.; Smith R. A.; Marangos J. P. Micrometer-thickness Liquid Sheet Jets Flowing in Vacuum. Rev. Sci. Instrum. 2017, 88, 083117 10.1063/1.4990130. 28863712

[ref31] Menzi S.; Knopp G.; Haddad A.; Augustin S.; Borca C.; Gashi D.; Huthwelker T.; James D.; Jin J.; Pamfilidis G.; et al. Generation and Simple Characterization of Flat Liquid Jets. Rev. Sci. Instrum. 2020, 91, 105109 10.1063/5.0007228. 33138597

[ref32] Werner W. S. M.; Smekal W.; Powell C. J. Simulation of Electron Spectra for Surface Analysis (SESSA), version 2.2; National Institute of Standard and Technology: Gaithersburg, MD, 2021.

[ref33] Lewis T.; Winter B.; Stern A. C.; Baer M. D.; Mundy C. J.; Tobias D. J.; Hemminger J. C. Dissociation of Strong Acid Revisited: X-ray Photoelectron Spectroscopy and Molecular Dynamics Simulations of HNO3 in Water. J. Phys. Chem. B 2011, 115, 9445–9451. 10.1021/jp205510q. 21688845

[ref34] Prisle N.; Ottosson N.; Öhrwall G.; Söderström J.; Dal Maso M.; Björneholm O. Surface/Bulk Partitioning and Acid/Base Speciation of Aqueous Decanoate: Direct Observations and Atmospheric Implications. Atmospheric Chemistry and Physics 2012, 12, 12227–12242. 10.5194/acp-12-12227-2012.

[ref35] Werner J.; Wernersson E.; Ekholm V.; Ottosson N.; Öhrwall G.; Heyda J.; Persson I.; Söderström J.; Jungwirth P.; Björneholm O. Surface Behavior of Hydrated Guanidinium and Ammonium Ions: A Comparative Study by Photoelectron Spectroscopy and Molecular Dynamics. J. Phys. Chem. B 2014, 118, 7119–7127. 10.1021/jp500867w. 24871810

[ref36] Suzuki Y.; Nishizawa K.; Kurahashi N.; Suzuki T. Effective Attenuation Length of an Electron in Liquid Water Between 10 and 600 eV. Phys. Rev. E 2014, 90, 010302 10.1103/PhysRevE.90.010302. 25122237

[ref37] Signorell R. Electron Scattering in Liquid Water and Amorphous Ice: A Striking Resemblance. Phys. Rev. Lett. 2020, 124, 205501 10.1103/PhysRevLett.124.205501. 32501058

[ref38] Ekimova M.; Quevedo W.; Szyc L.; Iannuzzi M.; Wernet P.; Odelius M.; Nibbering E. T. J. Aqueous Solvation of Ammonia and Ammonium: Probing Hydrogen Bond Motifs with FT-IR and Soft X-Ray Spectroscopy. J. Am. Chem. Soc. 2017, 139, 12773–12783. 10.1021/jacs.7b07207. 28810120

[ref39] Ekimova M.; Kubin M.; Ochmann M.; Ludwig J.; Huse N.; Wernet P.; Odelius M.; Nibbering E. T. J. Soft X-Ray Spectroscopy of the Amine Group: Hydrogen Bond Motifs in Alkylamine/Alkylammonium Acid-Base Pairs. J. Phys. Chem. B 2018, 122, 7737–7746. 10.1021/acs.jpcb.8b05424. 30024171

[ref40] Carter-Fenk K.; Head-Gordon M. On the Choice of Reference Orbitals for Linear-Response Calculations of Solution-Phase K-Edge X-Ray Absorption Spectra. Phys. Chem. Chem. Phys. 2022, 24, 26170 10.1039/D2CP04077H. 36278791

[ref41] Smith J. W.; Lam R. K.; Shih O.; Rizzuto A. M.; Prendergast D.; Saykally R. J. Properties of Aqueous Nitrate and Nitrite From X-Ray Absorption Spectroscopy. J. Chem. Phys. 2015, 143, 084503 10.1063/1.4928867. 26328852

[ref42] Söderström J.; Mårtensson N.; Travnikova O.; Patanen M.; Miron C.; Saethre L. J.; Bo̷rve K. J.; Rehr J. J.; Kas J. J.; Vila F. D.; et al. Nonstoichiometric Intensities in Core Photoelectron Spectroscopy. Phys. Rev. Lett. 2012, 108, 193005 10.1103/PhysRevLett.108.193005. 23003034

[ref43] Patanen M.; Travnikova O.; Zahl M. G.; Söderström J.; Decleva P.; Thomas T. D.; Svensson S.; Mårtensson N.; Bo̷rve K. J.; Sæthre L. J.; et al. Laboratory-Frame Electron Angular Distributions: Probing the Chemical Environment Through Intramolecular Electron Scattering. Phys. Rev. A 2013, 87, 4796–4808. 10.1103/PhysRevA.87.063420.

[ref44] Björneholm O.; Werner J.; Ottosson N.; Öhrwall G.; Ekholm V.; Winter B.; Unger I.; Söderström J. Deeper Insight into Depth-Profiling of Aqueous Solutions Using Photoelectron Spectroscopy. J. Phys. Chem. C 2014, 118, 29333–29339. 10.1021/jp505569c.

[ref45] Stener M.; Fronzoni G.; Toffoli D.; Decleva P. Time Dependent Density Functional Photoionization of CH_4_, NH_3_, *H*_2_*O*and HF. Chem. Phys. 2002, 282, 337–351. 10.1016/S0301-0104(02)00726-7. 16555887

[ref46] Novikovskiy N.; Sukhorukov V.; Artemyev A.; Demekhin P. Ab Initio Calculation of the Photoionization Cross Sections and Photoelectron Angular Distribution Parameters of CH_4_, NH_3_, *H*_2_*O*and CO. Eur. Phys. J. D 2019, 73, 79 10.1140/epjd/e2019-90628-8.

[ref47] Buttersack T.; Mason P. E.; McMullen R. S.; Martinek T.; Brezina K.; Hein D.; Ali H.; Kolbeck C.; Schewe C.; Malerz S.; et al. Valence and Core-Level X-Ray Photoelectron Spectroscopy of a Liquid Ammonia Microjet. J. Am. Chem. Soc. 2019, 141, 1838–1841. 10.1021/jacs.8b10942. 30673221 PMC6728086

