# Supplementary material for: Aqueous Ammonium Nitrate Investigated Using Photoelectron Spectroscopy of Cylindrical and Flat Liquid Jets
Source: J Phys Chem B. 2024 Jul 8;128(28):6866–75. doi: 10.1021/acs.jpcb.4c01755 (PMC11264267; doi:10.1021/acs.jpcb.4c01755)
Supplement: Supplementary file 1 — jp4c01755_si_001.pdf [file jp4c01755_si_001.pdf]

Supporting Information: Aqueous Ammonium Nitrate Investigated Using Photoelectron Spectroscopy of Cylindrical and Flat Liquid Jets

Tamires Gallo,[†,‡] Georgia Michailoudi,[¶,△] Joana Valerio,[§] Luigi Adriano,[§] Michael Heymann,[‖] Joachim Schulz,[§] Ricardo dos Reis Teixeira Marinho,[⊥,#] Flavia Callefo,[@] Noelle Walsh,[‡] and Gunnar Öhrwall[*,‡]

†*Synchrotron Radiation Research, Lund University, Box 118, SE-22100 Lund, Sweden*

‡*MAX IV Laboratory, Lund University, Box 118, SE-22100 Lund, Sweden*

¶*Nano and Molecular Systems Research Unit, University of Oulu, P.O. Box 3000, FI-90014 Oulu, Finland*

§*European XFEL, Holzkoppel 4, Schenefeld 22869, Germany*

‖*IBBS, Universität Stuttgart, Pfaffenwaldring 57, 70569, Stuttgart, Germany*

⊥*Institute of Physics, Brasilia University (UnB), 70.919-970, Brasília, Brazil*

#*Institute of Physics, Federal University of Bahia, 40.170-115, Salvador, BA, Brazil*

@*Brazilian Synchrotron Light Laboratory, LNLS, Brazilian Center for Research in Energy and Materials, CNPEM, CP 6192, 13085-970 Campinas, SP, Brazil*

△*Now at NOAA Chemical Sciences Laboratory, 325 Broadway, R/CSL6, Boulder, CO 80305, USA, and CIRES, University of Colorado Boulder, Boulder, CO 80309, USA*

E-mail: gunnar.ohrwall@maxiv.lu.se

Supporting Information Available

Further experimental details. Additional information and figures showing SESSA simulations,[S1] together with the applied parameters for the sample distribution and figures showing data fits using Igor Pro[S2] and the SPANCF curve fitting macro package, as presented in.[S3,S4] Tabulated data for N 1s peak area ratios NH_4^+:NO_3^-, for both cylindrical jet and flat jet delivery systems, and β values for NH_4^+ and NO_3^- derived assuming an isotropic background as well as an anisotropic background from SESSA simulations. Details regarding the derivation of the take-off angle dependence of the photoelectron signal ratios from MD simulations.

Further experimental details

The measurements were carried out on three different occasions: The investigation of the angular distribution with respect to the polarization using a cylindrical jet took place in March 2020, the measurements investigating the influence of the take-off angle using a flat-jet nozzle were performed in December 2022, and the measurements investigating the partial electron yield in "fixed mode" as a function of photon energy using a cylindrical jet were performed in June 2023.

Electrons ejected as a result of interaction with the synchrotron light passed through the opening of a skimmer cone positioned close to the liquid surface in the direction of the spectrometer. The nominal distance between the jet and the cone was 2 mm, with a cone opening of 0.5 mm, giving an opening angle of $\approx 7.15°$. This limited the angular acceptance of the spectrometer in the used kinetic energy range, in the non-dispersive as well as the dispersive direction. From numerical integration, we estimate that the finite acceptance of a cone with a half-angle opening of 7.15° will lead to an underestimate of $|\beta|$ values of $\approx 1.2\%$ when using Eq. (1). This is much less than the error bars we get from the statistical uncertainties and has, therefore, not been included in the analysis.

The sample used in all experimental campaigns was a 1.0 M (mol/dm^3) aqueous solution

of ammonium nitrate, prepared by dissolving NH_4NO_3 (Sigma Aldrich, $\geq$ 99.0% purity) in de-ionized water (MilliQ, 18.2 MΩcm). For the first measurements, the pH value for the solution was measured with pH sticks (MColorpHast, pH 0-14, Merck) and found to be approximately 5, well below the pK_a of NH_4^+ ($\approx$ 9.246[S5]) and above the pK_a of HNO_3 ($\approx$ -1.37[S5]). The content of ammonia and nitric acid is, therefore, expected to be negligible. For the subsequent measurements, the sample was prepared in the same way, and we expect a similar pH also in these cases. Before the measurements, each sample was filtered (Whatman Puradisc FP30 syringe filters, 1.2 µm) to remove solid particles.

The cylindrical liquid jet experiments in March 2020 were performed with a flow rate of 0.6 ml/min using a high-performance liquid chromatography (HPLC) pump (LabAlliance, Series III), with a pressure maintained in the range of 20 bar to 28 bar. For the cylindrical jet experiments in June 2023, a different HPLC pump was used (Knauer Blueshadow 40P), with a pressure maintained in the range of 3.2 bar to 3.4 bar for the same flow rate. For the flat jet experiment, the Knauer HPLC pump was used, and the liquid flow was set to 0.4 ml/min (pressure 62 bar - 63 bar). The He flow used in the flat jet experiments was set to 9.1 mg/min - 9.3 mg/min, as measured with a mass-flow meter (Bronkhorst F-111B). For all experiments, a degassing system installed in the liquid line (BIOTECH DEGASi PLUS Semi-Prep) was used in order to reduce the amount of dissolved gas and the risk of bubbles in the liquid flow.

For the cylindrical jet experiments in March 2020, N 1s core level spectra were recorded at 90° and at 54.7° relative to the horizontal polarization of the synchrotron radiation, at photon energies from 470 eV to 530 eV, in steps of 10 eV. Data was first recorded at 54.7° for all energies, after which the angle was changed to 90°, and the second set of data was recorded using the same experimental conditions. The probed volume changes between the two angles and the transmission of the spectrometer may also be affected, but as explained in the main text, we have relied on the background intensity to compare intensities between the data sets. The background electrons are created in the same volume as the N 1s electrons,

and as they have nearly the same kinetic energy as the N 1s electrons, they will be affected by the same transmission function and should, therefore, provide a reasonable means of normalization. The beamline exit slit opening for these measurements was 100 µm, giving photon bandwidths of ≈ 190 meV -230 meV over this range. The analyzer was operated with a pass energy of 200 eV, and a 500 µm curved slit was used, resulting in a spectrometer energy resolution of 250 meV.

For the cylindrical jet experiments in June 2023, the spectrometer was oriented with the lens axis at 54.7° with respect to the polarization vector of the radiation. In this case, the analyzer was operated with a 1.5 mm straight slit and a pass energy of 200 eV, resulting in a spectrometer energy resolution of 750 meV. Considering the large separation of the two N 1s peaks, this lowering of the resolution is no limitation in the analysis of the data. The beamline exit slit opening was 100 µm also for these recordings.

For the experiments with the flat jet nozzle, a photon energy of 500 eV was used, and the angle between the polarization vector of the light and the spectrometer was set to 90°. The beamline exit slit was opened to 50 µm, giving a photon bandwidth of ≈ 100 meV, and the same spectrometer settings as used for the cylindrical jet experiment were implemented. The take-off angle was estimated by observing the angle at which the first liquid sheet was narrowest (viewed with a camera arranged co-linear with the spectrometer lens axis, defined as 0°) and then the angular scale on the rotary stage was used to set the angle. We estimate that this procedure will give an uncertainty of ± 5° in the take-off angle, which has been included in the presentation of the data.

The experimental N 1s photoelectron spectra were fitted with symmetric Voigt functions, using Igor Pro[S2] and the SPANCF curve fitting macro package, as presented in.[S3,S4] In the fits, the Lorentzian lifetime width was fixed to 0.14 eV (similar to that found for N 1s photoemission in N_2,[S6] and HCN[S7]) and the Gaussian widths were allowed to vary but were constrained to have the same value at the two angles for each photon energy. In the spectral fits, a linear background and a weak Gaussian peak at lower kinetic energy (to account for

inelastic loss structures in the spectrum) were included. Example fits for the cylindrical and flat jet experiments are presented in Figs. **S3**, **S5**, and **S6**. The binding energy calibration of the spectra was performed versus that of the HOMO in liquid water, at 11.16 eV.[S8] The SPANCF fitting routine estimates the standard deviation of the fitting parameters from the curvature matrix of the χ^2 surface, and we have used these values to derive errors for the peak areas. We have only included statistical contributions in the error analysis, and the error bars presented in the figures below are derived as $\pm$ 1.96 times the compounded error from the calculations based on the peak areas from the fits.

SESSA simulations

We have used the software program SESSA (Simulation of Electron Spectra for Surface Analysis)[S1] to simulate electron spectra from aqueous solutions of NH_4NO_3 for all photon energies. The aim of these simulations was not to derive information about the N 1s photoelectron peaks but to learn about the background created by inelastically scattered electrons from valence orbitals and N 1s Auger transitions from molecules and ions in the solution. Simulations for the angles 90° and 54.7° provided information about the angular anisotropy of the background, which we have used in a normalization procedure to derive values for the β parameter of the N 1s peaks. In the simulations, the β values and binding or kinetic energies for the photoelectron and Auger lines come from tabulated atomic data. The N 1s photoelectrons from both NH_4^+ and NO_3^- were thus assumed to have the same binding energy and the same anisotropy parameter value (β = 2, independent of photon energy). The N 1s data for the two ions in the simulations can, therefore, not be distinguished, and, consequently, not be immediately compared to the experimental results.

SESSA simulations were performed for the photon energies 530, 520, 510, 500, 490, 480, and 470 eV. Since SESSA offers no possibility of simulations for a cylindrical surface, a planar surface was used in our simulation. To emulate the variation in the take-off angle

from the cylindrical surface of the jet, the angle between the planar sample surface and the spectrometer was varied from 0° to 100° in steps of 5°. The spectrometer was assumed to have a conical acceptance with a half-angle 12° (slightly larger than the real acceptance). Due to this finite acceptance angle, some intensity is still seen in the simulations beyond the 90° take-off angle. The polarization vector for the incident light was set to have an angle of 54.7° and 90° relative to the spectrometer. Example spectra for the case with photon energy 530 eV are shown in Fig. **S1**.

The sample's Relative Surface Area (RSA) was set to 1.005, to emulate the surface roughness of the liquid-vapor interface. To investigate the possible influence of variations in the surface concentration of the solute, we used a 3-layer description of the sample: a 2 Å thick top-most layer containing water molecules, a 5 Å thick layer with varying NH_4NO_3 concentration (1, 2, or 3 mol%, approximately corresponding to 0.5, 1.0, and 1.5 mol/kg), and a bulk substrate with 2 mol% (≈ 1 mol/kg) of NH_4NO_3 in water, see Table S1. The densities used in the simulations were taken from Ref.[S9] Additionally, we simulated a sample with 2 mol% (≈ 1 mol/kg) of NH_4NO_3 in water for all 3 layers. The spectral difference in the region of interest for the applied simulation parameters for these sample variations is relatively small. The simulations thus show that possible density variations in the surface region will only have a small influence on the results for the background.

Table S1: Sample parameters used in the SESSA simulations; a 3-layer model with pure water at the surface, 1 mol% NH_4NO_3 for an intermediate layer below the surface, and 2 mol% NH_4NO_3 for the bulk

Command	Input	Layer
SAMPLE SET MATERIAL	/H2/O/	LAYER 1
SAMPLE SET DENSITY	1.000e00	LAYER 1
SAMPLE SET THICKNESS	2	LAYER 1
SAMPLE SET MATERIAL	(/H2/O/)99(/N2/O3/H4/)1	LAYER 2
SAMPLE SET DENSITY[S9]	1.034e00	LAYER 2
SAMPLE SET THICKNESS	5	LAYER 2
SAMPLE SET MATERIAL	(/H2/O/)98(/N2/O3/H4/)2	LAYER 3
SAMPLE SET DENSITY[S9]	1.034e00	LAYER 3

Figure **S1**: Spectra generated using a model with 2 mol% of NH_4NO_3 in layer 2 and layer 3, using a photon energy of 530 eV, and for sample angles from 0° to 100° in steps of 5° at lens axis angle (a) 54.7°, (b) 90°. Figures (c) and (d) show a zoom of spectra at 54.7° and 90°, respectively, for the region around the N 1s core level between 60 to 180 eV.

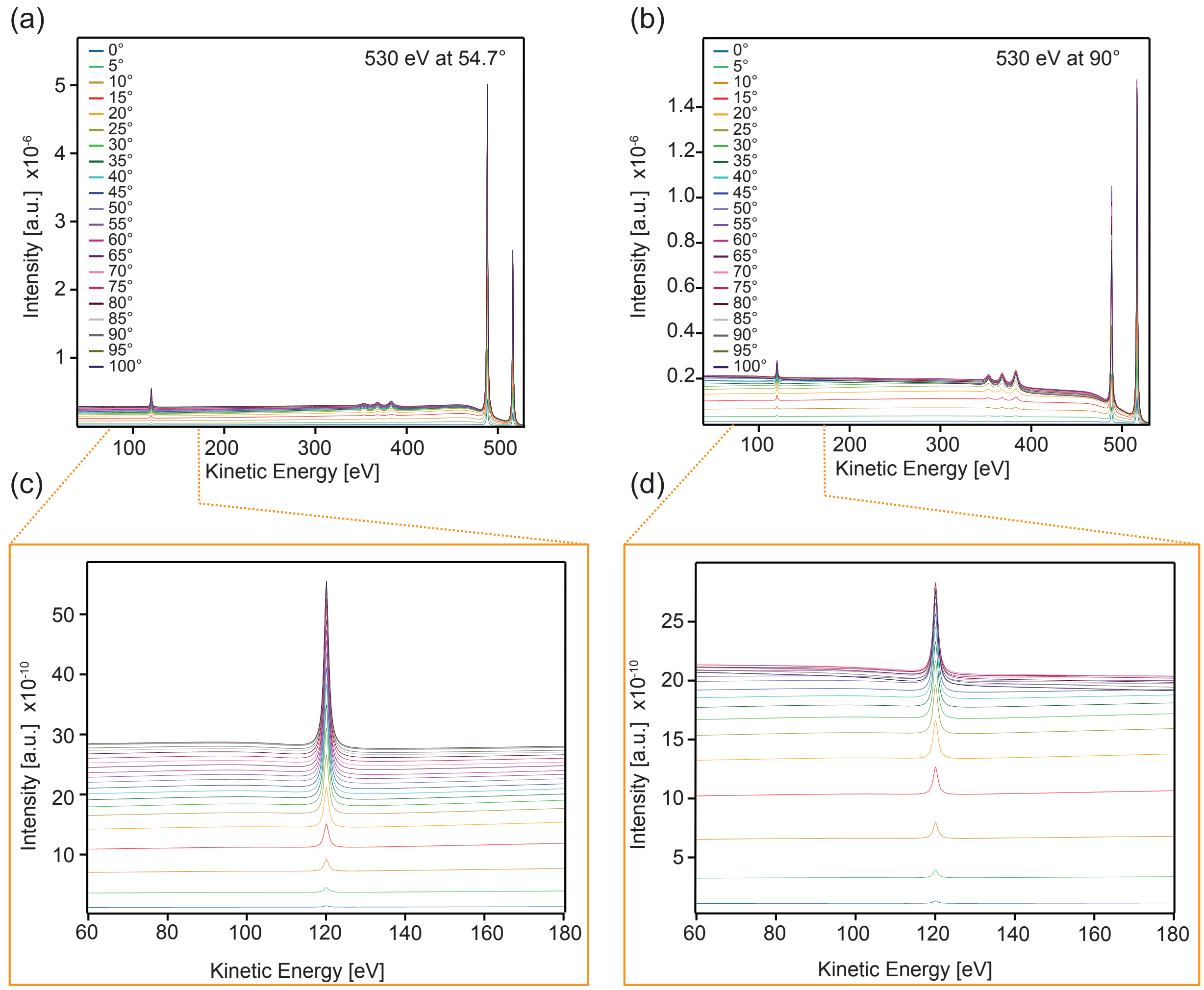

Fig. **S2**(a) shows the average of all simulated take-off angles exhibited in Fig. **S1** (photon energy 530 eV), for the lens axis angles 54.7° and 90° with respect to the polarization vector. The same approach was taken for all energies between 470 to 530 eV. In order to establish the angular anisotropy of the background at each photon energy, we quantified the background in the vicinity of the N 1s line by determining the intensity at one point 10 eV below the N 1s line, and at one point 10 eV above the N 1s line, see figure **S2**(b). From those values, we calculated the ratio of the backgrounds at 54.7° and 90° above and below the N 1s for all

Figure **S2**: (a) Spectra generated using an average of the spectra in figure **S1** for lens axis angles 54.7° and 90°. (b) Zoom for N 1s core level between 60 to 180 eV, indicating the points used for the background ratio calculations (N 1s core level ± 10 eV).

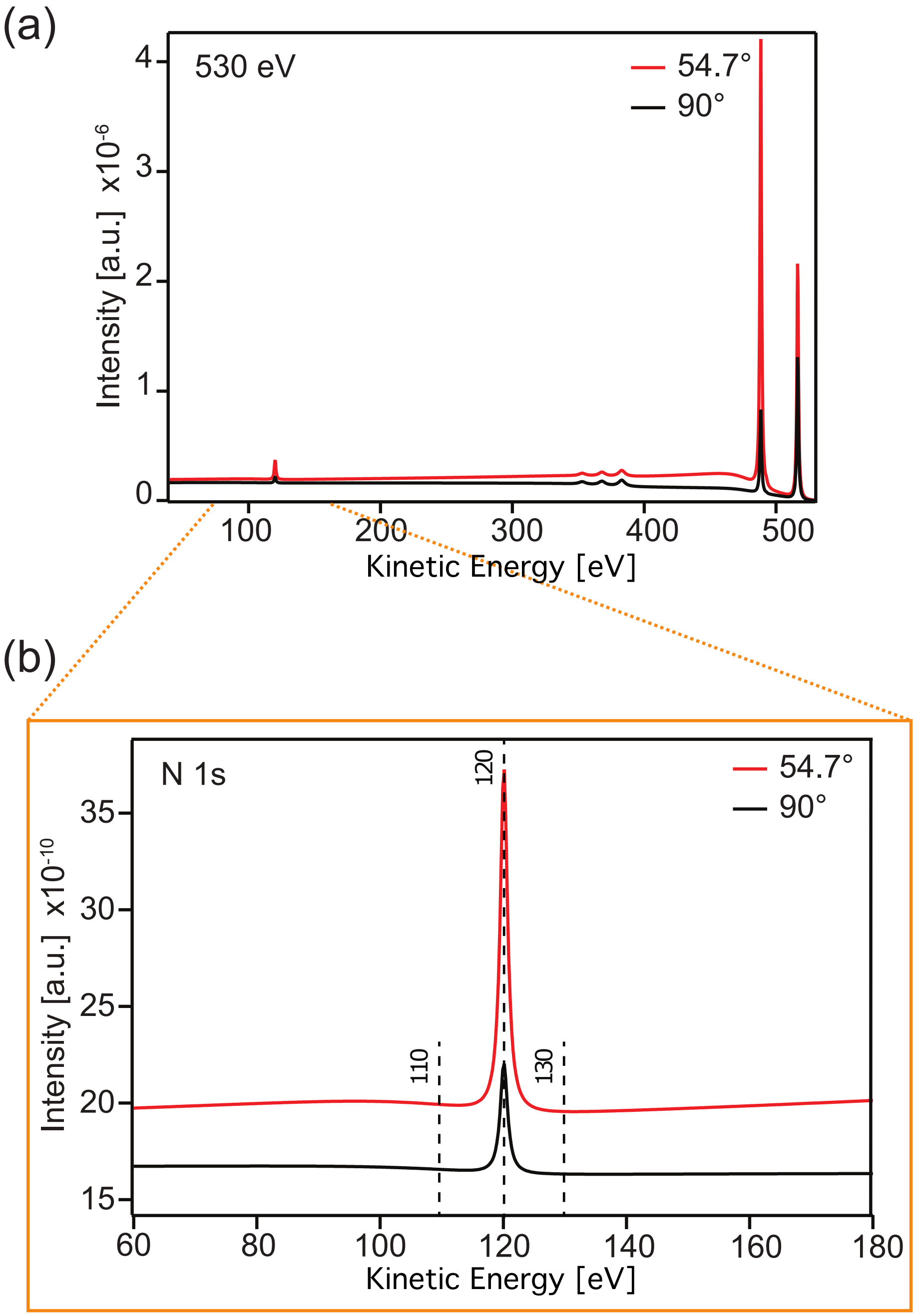

photon energies, and the average was used in the calculation of the β value. The ratio varies from 1.19 to 1.20 between 470 and 530 eV for the case with 2 mol% of NH_4NO_3 in layers 2 and 3, corresponding to a β value of $\approx 0.32 - 0.33$. The difference between the different concentration models was very small, much smaller than statistical uncertainties and other errors, so we used the case with 2 mol% of NH_4NO_3 in the second and third layer for the calculation of the β values.

Experimental data fitting

Fig. **S3** shows fits of the experimental spectra recorded with a cylindrical nozzle, with the lens axis angles 54.7° and 90° with respect to the polarization vector, for the photon energy 490 eV. Note that the relative intensity of the NH_4^+ peak decreases relative to the NO_3^- peak at 90°. The data are the same as those shown in Fig. 2 in the main text.

Fig. **S4** shows the measured peak areas of the NH_4^+ and NO_3^- features, normalized to acquisition time (number of sweeps) as a function of photon energy, for lens axis angles 54.7° and 90° with respect to the polarization vector. The calculated cross section for atomic N 1s photoionization[S10,S11] is also included for comparison. The measured intensities depend on many experimental factors, for instance, the alignment of the photon beam and liquid jet, and the transmission of the spectrometer; we have therefore chosen to rely on the ratio of the intensities, which is a more robust quantity, in our analysis. The included error bars only reflect the statistical uncertainty. However, note that the NH_4^+ intensity at 54.7° monotonically decreases in a relatively similar way to the calculated cross-section, whereas the NO_3^- intensity has an oscillatory behavior with a maximum of around 490 eV. This behavior can be compared to that shown in Fig. 4 c) in the main text, containing raw data for "fixed" mode measurements.

Fig. **S5** a) shows experimental spectra recorded in "fixed" mode, for the lens axis angle 54.7° with respect to the polarization vector, using the photon energy 495 eV, for 1.0 M

Figure **S3**: Example fit of the N 1s core level of NH_4NO_3 at 490 eV photon energy at (a) 54.7°, and (b) 90°.

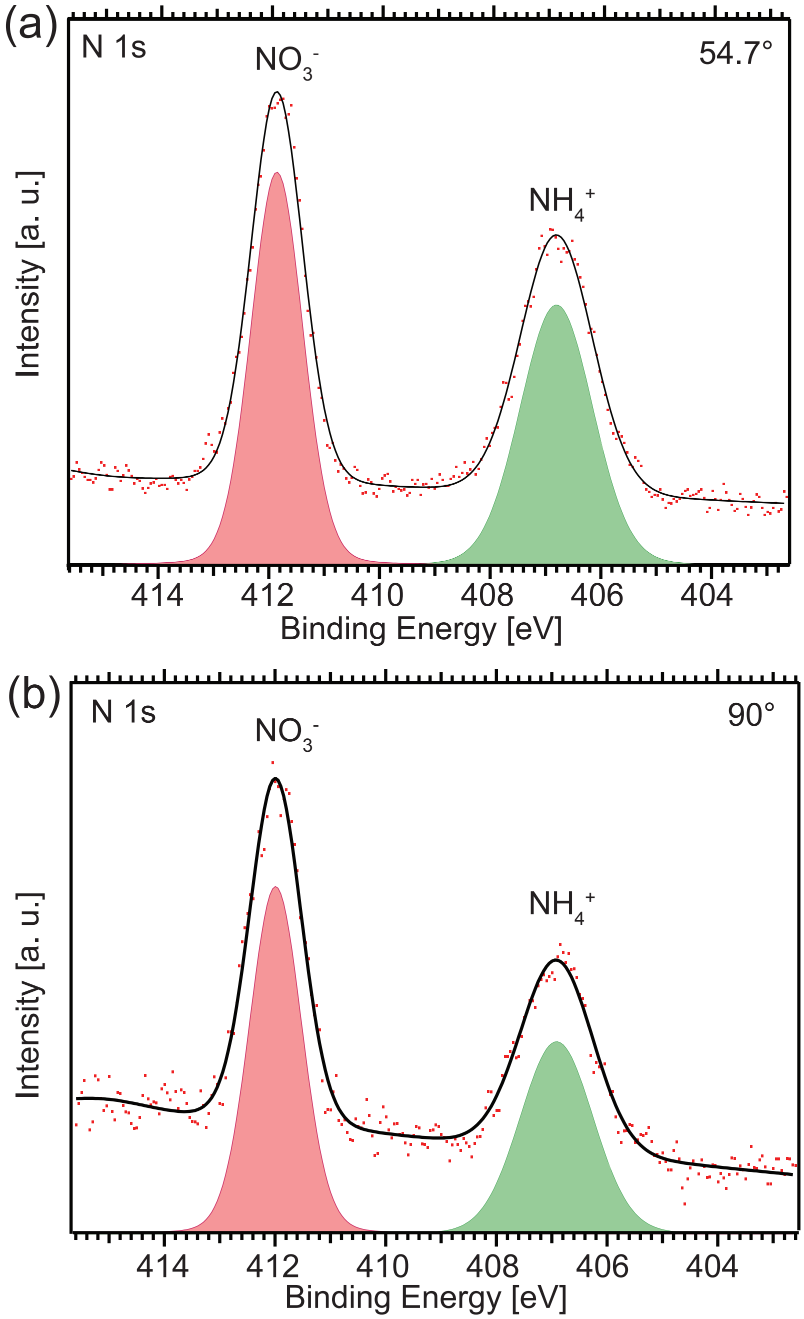

Figure **S4**: Peak areas of the N 1s lines from NH_4^+ (blue symbols) and NO_3^- (red symbols), recorded at 54.7° (filled circles) and 90° (filled lozenges). The calculated cross section for N 1s photoionization (black solid line, right scale) is also included.

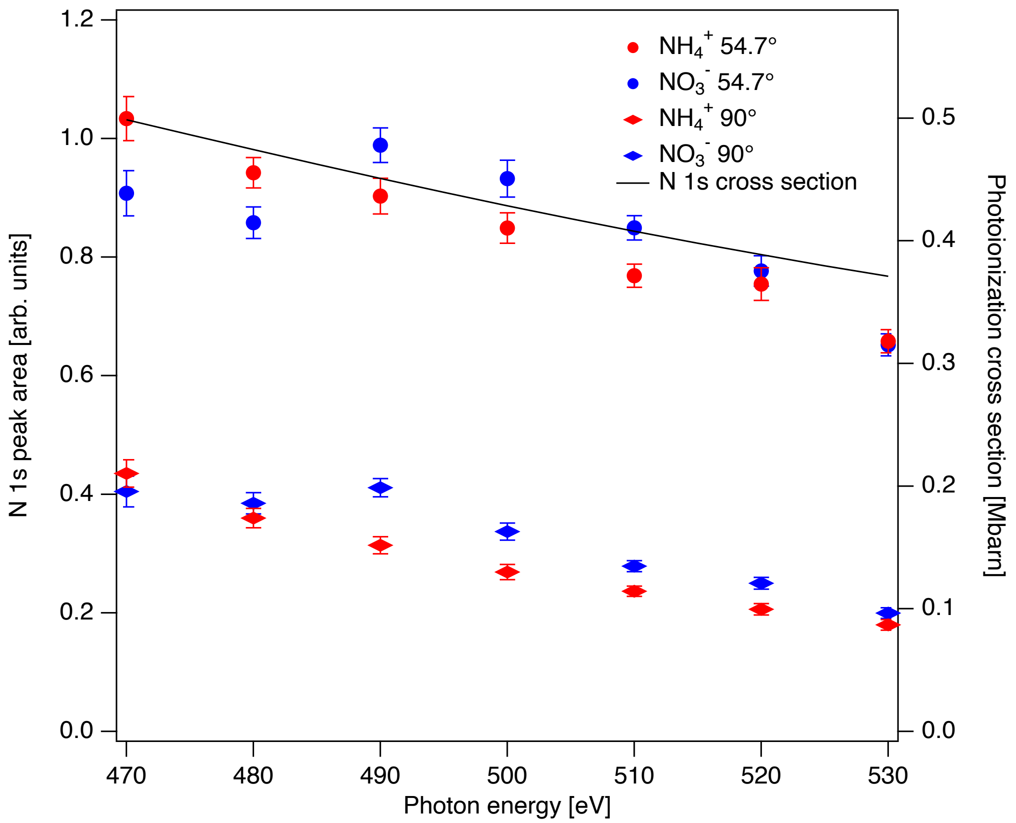

NH_4NO_3 and 25 mM NaCl aqueous solutions. The data are the same as those shown in the maps in Fig. 4 a) and b) in the main text. As seen, the background intensity varies across the detected range, mostly due to variation in transmission for the range of kinetic energies reaching the detector, and the inhomogeneity in detector response. In b), a spectrum obtained by normalizing the NH_4NO_3 data by the NaCl data is shown, where, as expected, the background intensity is more flat, and the intensity of the NH_4^+ peak relative to the NO_3^- peak has increased compared to the spectrum in a). A fit of the data is also included.

Fig. **S6** shows fits of the experimental spectra recorded at 500 eV with a flat jet nozzle, with the spectrometer rotated to 90° with respect to the polarization vector, for two take-off angles. The relative intensity of the two peaks is independent of the take-off angle, indicating a similar distribution of the two ions in the surface region.

Estimate of intensity ratio as a function of take-off angle from MD result

We obtained the density profile for NO_3^- and the charge density profile for the case of a 2.5 mol/kg solution of NH_4NO_3 from the MD simulations presented in Fig. 8 of ref.,[S12] specifically those calculated using the optimized potential for liquid potentials with electronic continuum correction model (OPLS/ECC). We digitized the values from the graph using an online tool,[S13] and the number density profile of NH_4^+ was then calculated from these two data sets rather than digitized directly from the plot due to its small scale in the figure (derived concentrations in mol/dm^3 shown in Fig. **S7**). The density profiles were plotted to 1.4 nm below the surface in ref.[S12] We extrapolated the data to 10 nm from the values at 1.4 nm by assuming a constant concentration value for NO_3^- below 1.4 nm and a linearly varying value for NH_4^+ between 1.4 and 2.6 nm, to reach equal and constant concentrations below 2.6 nm, which gives a stoichiometric ratio of the two ions for the total volume. As can be deduced from the plot, the concentration is higher than 2.5 mol/kg in the interior.

Figure **S5**: a) Example spectra for 1.0 M NH_4NO_3 and 25 mM NaCl aqueous solutions, recorded in the so-called "fixed" mode (see main text) at 495 eV photon energy (from map presented in Fig. 4 of the main text). The intensity varies across the detected range due to the kinetic energy dependence of transmission and the detector's inhomogeneity. b) Spectrum where the NH_4NO_3 data has been normalized to the NaCl data. A fit of the data is also inlcuded.

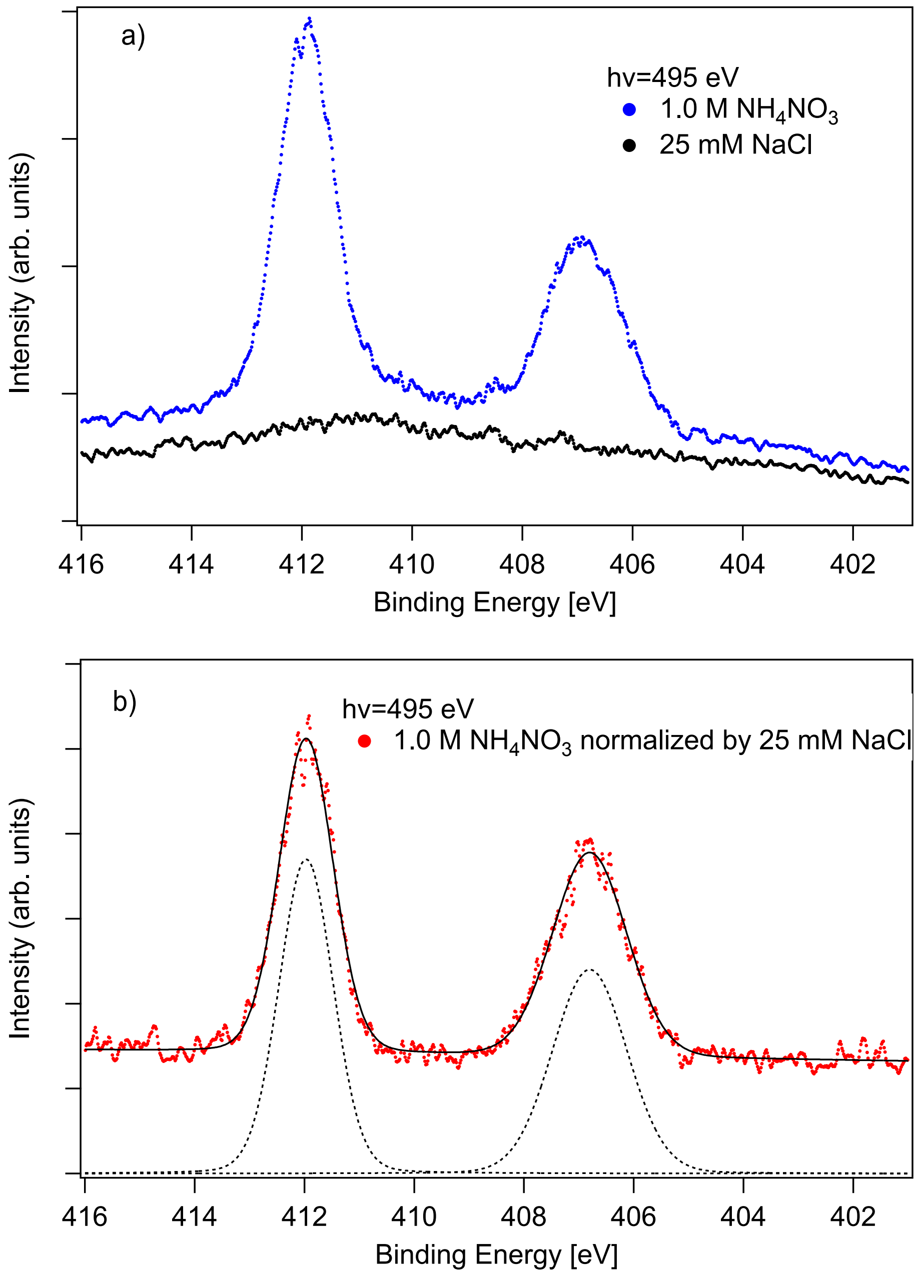

Figure **S6**: Example spectra (blue and red symbols), together with fits (solid black lines), for N 1s photoelectron spectra recorded with a flat jet nozzle at 500 eV, for two take-off angles (10° and 80°).

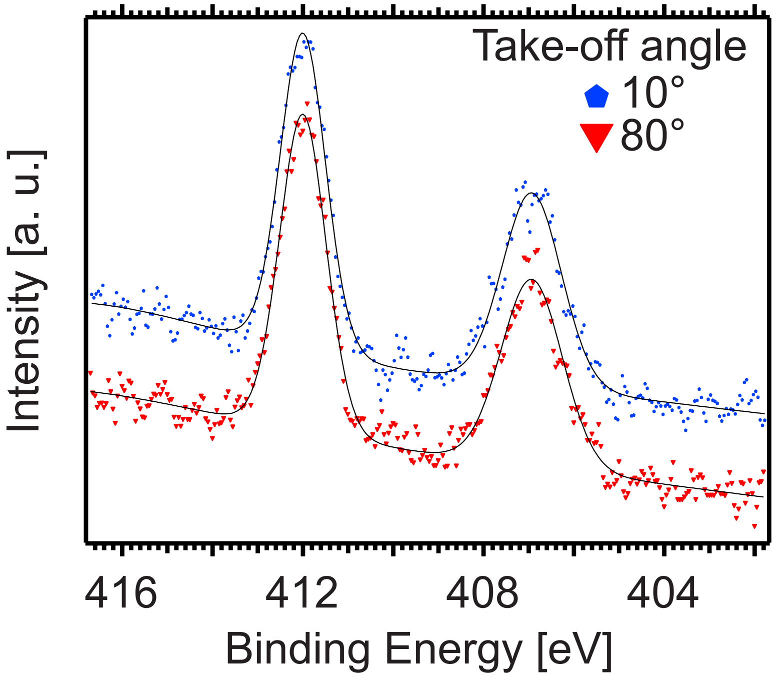

This is a consequence of the finite extension of the MD simulation, which used a slab of 4 nm thickness,[S12] i.e., a maximum distance to the interface of 2 nm, and thus an interior concentration higher than the average (2.5 mol/kg). As we are only interested in the ratios of the NH_4^+ and NO_3^- signals, only the relative difference in the distribution and not its absolute value will affect the comparison.

The intensity of the electron signal (related to each ion) as a function of the take-off angle was calculated for both ions as a numeric integration of the number density of the ions as a function of the distance from the surface, in steps of 0.01 nm down to 10 nm, dampened by the inelastic scattering. A finite spectrometer acceptance of $\pm$ 7° was also considered (similar to that of the experiment, see Experimental details above), by summing the intensities within this range around every angle. The summation was performed down to 1° at the lower edge and up to 90° at the higher edge of the take-off angle range. The ratio of the calculated intensities was then determined, and (arbitrarily) normalized to coincide with the average of the experimental data at 90°. We have calculated values for effective attenuation lengths (EAL) of 1.0 nm and 1.5 nm, similar to experimentally obtained values for the $\approx$ 90 eV kinetic energy electrons.[S14]

Figure **S7**: Concentrations of NH_4^+ (red solid line) and NO_3^- (blue solid line) in the surface region of a 2.5 mol/kg NH_4NO_3 aqueous solution (zero at Gibbs dividing plane), derived by digitizing the data from the MD simulations using the OPLS/ECC model presented in Fig. 8 of ref.[S12]

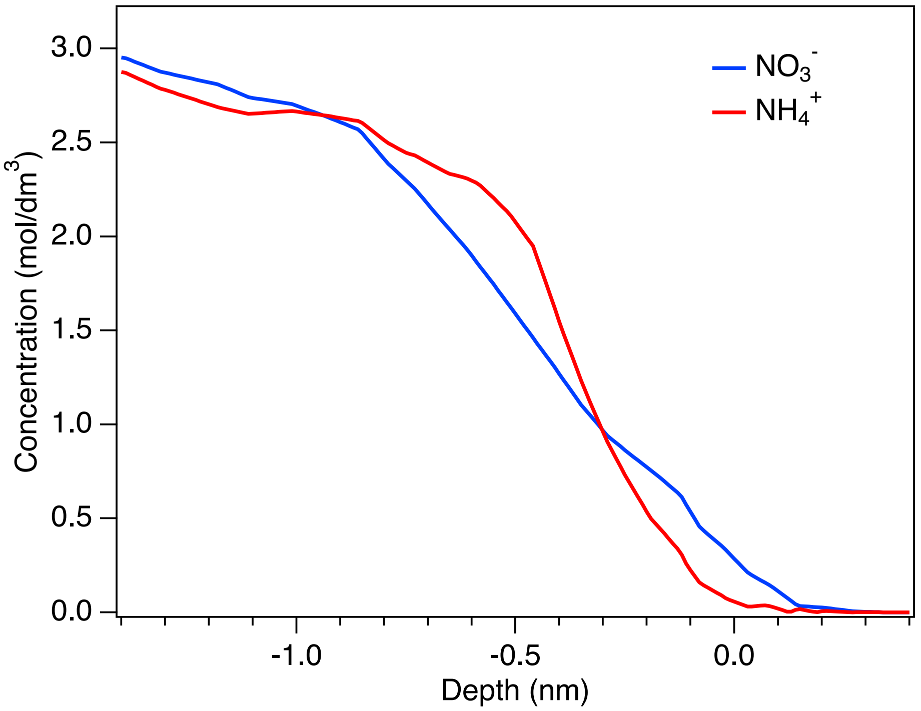

Table S2: Experimentally obtained data derived from N 1s peak areas using the cylindrical jet delivery system. (a) N 1s ratio of NH_4^+:NO_3^- at 54.7°, (b) error for NH_4^+:NO_3^- at 54.7° (σ*1.96), (c) N 1s ratio of NH_4^+:NO_3^- at 90°, (d) error for NH_4^+:NO_3^- at 90° (σ*1.96), (e) β values for NH_4^+ assuming an isotropic background, (f) error β NH_4^+ isotropic background (σ*1.96), (g) β values for NO_3^- assuming an isotropic background , (h) error β NO_3^- isotropic background (σ*1.96), (i) β values for NH_4^+ assuming an anisotropic background, (j) error β NH_4^+ anisotropic background (σ*1.96), (k) β values for NO_3^- assuming an anisotropic background, (l) error β NO_3^- isotropic background (σ*1.96).

NH_4^+:NO_3^-	54.7°		90°	
hν (eV)	a	b	c	d
470	1.139	0.063	1.076	0.089
480	1.098	0.045	0.935	0.061
490	0.913	0.041	0.764	0.045
500	0.912	0.041	0.799	0.051
510	0.905	0.032	0.848	0.042
520	0.974	0.048	0.855	0.053
530	1.010	0.042	0.901	0.060
β isotropic bgr	NH_4^+		NO_3^-	
hν (eV)	e	f	g	h
470	1.105	0.071	1.053	0.080
480	1.175	0.062	1.031	0.058
490	1.251	0.071	1.104	0.053
500	1.283	0.072	1.182	0.064
510	1.297	0.057	1.250	0.052
520	1.349	0.080	1.259	0.066
530	1.357	0.079	1.280	0.067
β anisotropic bgr	NH_4^+		NO_3^-	
hν (eV)	i	j	k	l
470	1.248	0.080	1.204	0.092
480	1.307	0.069	1.186	0.067
490	1.372	0.078	1.249	0.060
500	1.400	0.079	1.316	0.071
510	1.413	0.062	1.373	0.057
520	1.456	0.086	1.380	0.072
530	1.465	0.085	1.400	0.074

Table S3: Experimentally obtained N 1s peak area ratios NH_4^+:NO_3^- as a function of take-off angle using the flat-jet delivery system. The photon energy was hν= 500 eV, the angle between the polarization vector and the lens axis was θ=90°.

Take-off angle (°)	NH_4^+:NO_3^-	Error (σ*1.96)
10	0.781	0.066
24	0.804	0.062
38	0.811	0.058
52	0.753	0.064
66	0.786	0.072
80	0.767	0.066
80	0.772	0.058

References

(S1) Werner, W. S. M.; Smekal, W.; Powell, C. J. *Simulation of Electron Spectra for Surface Analysis (SESSA)*, version 2.2; National Institute of Standard and Technology: Gaithersburg, MD, 2021.

(S2) *Igor Pro*, version 8.04; WaveMetrics, Inc.: Lake Oswego, OR, 2019.

(S3) Kukk, E.; Snell, G.; Bozek, J. D.; Cheng, W.-T.; Berrah, N. Vibrational Structure and Partial Rates of Resonant Auger Decay of the N1 $\vec{s}$ 2π Core Excitations in Nitric Oxide. *Phys. Rev. A* **2001**, *63*, 062702.

(S4) Kukk, E.; Ueda, K.; Hergenhahn, U.; Liu, X.-J.; Prümper, G.; Yoshida, H.; Tamenori, Y.; Makochekanwa, C.; Tanaka, T.; Kitajima, M.; Tanaka, H. Violation of the Franck-Condon Principle due to Recoil Effects in High Energy Molecular Core-Level Photoionization. *Phys. Rev. Lett.* **2005**, *95*, 133001.

(S5) Speight, J. G. *Lange's Handbook of Chemistry*, 16th ed.; McGraw-Hill Education: New York, Chicago, San Francisco, Lisbon, London, Madrid, Mexico City, Milan, New Delhi, San Juan, Seoul, Singapore, Sydney, Toronto, 2005; https://www.accessengineeringlibrary.com/content/book/9780071432207.

(S6) Neeb, M.; Rubensson, J. E.; Biermann, M.; Eberhardt, W. Coherent Excitation of Vibrational Wave Functions Observed in Core Hole Decay Spectra of O_2, N_2 and CO. *Journal of Electron Spectroscopy and Related Phenomena* **1994**, *67*, 261–274.

(S7) Giertz, A.; Børve, J. K.; Bässler, M.; Wiesner, K.; Svensson, S.; Karlsson, L.; Sæthre, L. J. Vibrationally Resolved Photoelectron Spectra of the Carbon 1s and Nitrogen 1s Shells in Hydrogen Cyanide. *Chemical Physics* **2002**, *277*, 83–90.

(S8) Winter, B.; Weber, R.; Widdra, W.; Dittmar, M.; Faubel, M.; Hertel, I. V. J. Full

Valence Band Photoemission from Liquid Water Using EUV Synchrotron Radiation. *Journal of Physical Chemistry A* **2004**, *108*, 2625–2632.

(S9) Perry, R. H.; Green, D. W.; Maloney, J. O. *Perry's chemical engineers' handbook*, 7th ed.; R. R. Donnelley and Sons Company.: McGraw-Hill Companies, Inc., 1997.

(S10) Yeh, J. J. *Calculation of Photoionization Cross-Sections and Asymmetry Parameters*; R. R. Donnelley and Sons Company.: Langhorne, PE (USA), 1993.

(S11) Yeh, J. J.; Lindau, I. Atomic Subshell Photoionization Cross Sections and Asymmetry Paramters: $1 \leq Z \leq 103$. *Atomic Data and Nuclear Data Tables* **1985**, *32*, 1–155.

(S12) Mosallanejad, S.; Oluwoye, I.; Mohammednoor, A.; Gore, J.; Dlugogorski, B. Z. Interfacial and Bulk Properties of Concentrated Solutions of Ammonium Nitrate. *Physical Chemistry Chemical Physics* **2020**, *22*, 12227–12242.

(S13) PlotDigitizer. https://plotdigitizer.com (accessed January 21, 2023).

(S14) Thürmer, S.; Seidel, R.; Faubel, M.; Eberhardt, W.; Hemminger, J. C.; Bradforth, S. E.; Winter, B. Photoelectron Angular Distributions from Liquid Water: Effects of Electron Scattering. *Physical Review Letters* **2013**, *111*, 173005.
